# Using Smartphone GPS Data to Detect the Risk of Adolescent Suicidal Thoughts and Behaviors

**DOI:** 10.1001/jamanetworkopen.2024.56429

**Published:** 2025-01-27

**Authors:** Randy P. Auerbach, Paul A. Bloom, David Pagliaccio, Ranqing Lan, Hanga Galfalvy, Alma Bitran, Katherine Durham, Ryan Crowley, Karla Joyce, Ashley Blanchard, Lauren S. Chernick, Peter S. Dayan, Julia Greenblatt, Lauren E. Kahn, Giovanna Porta, Trinity C. Tse, Jeffrey F. Cohn, Louis-Philippe Morency, David A. Brent, Nicholas B. Allen

**Affiliations:** 1 Department of Psychiatry, Columbia University, New York, New York; 2 New York State Psychiatric Institute, New York; 3 Department of Psychology, University of Oregon, Eugene; 4 Department of Psychiatry, University Pittsburgh Medical Center, Pittsburgh, Pennsylvania; 5 Department of Emergency Medicine, Columbia University, New York, New York; 6 Department of Psychology, University of Pittsburgh, Pennsylvania; 7 Language Technology Institute, Carnegie Mellon University, Pittsburgh, Pennsylvania

## Abstract

**Importance:**

Suicide rates among adolescents continue to rise, but there are a lack of clinical tools to predict when youths may be at risk for suicidal behaviors.

**Objective:**

To identify whether geolocation metrics, assessed through an app installed on adolescents’ personal smartphones, could detect the risk of next-week suicidal events and clinically meaningful suicidal ideation.

**Design, Setting, and Participants:**

This case series study included high-risk adolescents aged 13 to 18 years reporting a current affective and/or substance use disorder, oversampled for suicidal thoughts and behaviors (STB). Participants were recruited from the greater New York City and Pittsburgh communities through psychiatric outpatient programs, emergency departments, medical center research registries, and social media. Participants installed the Effortless Assessment Research System (EARS) software application onto their personal smartphones, which obtained passive sensor data, including geolocation metrics (via the global positioning system [GPS]), as well as weekly experience sampling data probing STB for the duration of the 6-month study. Adolescents also completed clinical assessments at baseline as well as during the 1-, 3-, and 6-month follow-up assessments. Statistical analysis was performed from March 2023 to November 2024.

**Main Outcomes and Measures:**

Repeated measures mixed-effects logistic models estimated whether weekly aggregates of geolocation features (ie, entropy, homestay, distance traveled) were associated with next-week suicidal events (ie, suicide attempts, psychiatric hospitalizations, emergency department visits for suicide concerns) and clinically meaningful ideation (via weekly experience sampling).

**Results:**

Overall, 186 participants were included in this study (148 [79.6%] female; 19 [10.2%] Asian, 23 [12.4%] Black, and 106 [57.0%] White), with a mean (SD) age of 16.4 (1.7) years. Greater homestay (amount of time spent at home) on a given week, relative to one’s own mean, was associated with 2-fold greater odds of suicidal events during the subsequent week (odds ratio, 1.99 [95% CI, 1.15-3.45]). Results were not significant for entropy and distance traveled metrics. However, using leave-future-out validation, the accuracy of the homestay model was modest (area under the receiver operating characteristic curve, 0.64 [95% CI, 0.50-0.78]).

**Conclusions and Relevance:**

Advancements in smartphone technology afford unique opportunities to capture affective and behavioral dynamics that presage suicide risk. This case series study found that greater homestay obtained through smartphone GPS data over the course of a week, relative to one’s own mean, was associated with greater odds of a suicidal event in the subsequent week. Although accuracy was modest, these findings offer a novel starting point for suicide prevention research, particularly as smartphone sensor data may have the capacity to identify who is at risk while also providing an opportunity to deliver clinical tools when that risk is greatest.

## Key Points

**Question:**

Can passive sensor data from adolescent smartphones identify increased risk of suicidal events (ie, attempt, emergency department visit, and/or psychiatric hospitalization) among high-risk youths over a 6-month period?

**Findings:**

This case series study of 186 high-risk adolescents found that greater homestay (amount of time spent at home) obtained through smartphone geolocation data over the course of a week, relative to one’s own mean, was associated with 2-fold greater odds of a suicidal event in the subsequent week.

**Meaning:**

These results suggest that with further validation, naturalistic use of personal smartphones has potential to detect short-term risk of suicidal events, which may facilitate the delivery of timely suicide prevention strategies.

## Teaser

This case series study examines whether an app installed on adolescents’ personal smartphones could identify risk of next-week suicidal events and clinically meaningful suicidal ideation by using geolocation metrics via global positioning system (GPS) data.

## Introduction

Suicide rates continue to rise among adolescents,^1^ and although there are clinical indications regarding which youths may be at risk of hurting themselves, we do not currently possess tools that clarify when suicide risk is greatest.^2^ Real-time monitoring approaches, including experience sampling methods (ESM) and mobile sensor monitoring, afford unique access to dynamic affective and behavioral changes in adolescents’ lives.^3,4^ ESM studies have addressed foundational issues clarifying the time scale of ideation,^5^ identifying high-risk profiles,^6^ and recently, delineating potential thresholds at which to intervene.^7^ Less research, however, has demonstrated the utility of passive sensor data approaches collected naturalistically and unobtrusively via personal smartphones for the prediction of suicidal thoughts and behaviors (STB). This innovative approach has enormous potential, as 95% of adolescents own smartphones.^8^ Furthermore, youths may be less inclined to report their affect and behaviors during acute distress, which may explain highly variable ESM response rates among youths.^9^ Promisingly, our work found that suicide acuity among adolescents was unrelated to patterns of missingness for mobile sensor data^10^ and leveraging smartphone data to predict suicidal events would reflect a paradigm-shifting approach for suicide prevention.

Increasingly, global positioning system (GPS) data from smartphones have been used to detect depressive states. Geolocation sensors provide key insights about behavioral changes that presage risk, including homestay (ie, time spent in one’s home), distance traveled (ie, time outside the home and energy exerted), and entropy (ie, variability of the pattern of places visited). Greater homestay and reduced entropy are associated with depressive symptom severity,^11,12^ whereas reduced distance traveled differentiates participants with a lifetime history of depression from healthy individuals.^13^ Together, this work has demonstrated that mobility patterns may reflect clinically meaningful states relating to social connectedness, behavioral withdrawal, and anhedonia factors directly related to youth suicide risk.^14,15,16^ Furthermore, recent work has shown potential for using smartphone mobility measures in predicting STB among adults,^17^ although the capacity of these measures to detect adolescent suicide risk remains unclear.

To improve the short-term prediction of STB, high-risk adolescents were followed over a 6-month period. Mobility patterns were tracked through a smartphone app, and weekly ESM probed the frequency of suicidal ideation as well as the presence of suicide plans and attempts. Suicidal events (eg, attempts, emergency department visits, hospitalizations) also were assessed at the 1-, 3-, and 6-month follow-up assessments. Given prior work in depression,^11,12,13^ we hypothesized that greater homestay as well as reduced entropy and distance traveled on a given week would be associated with next-week clinically meaningful suicidal ideation and suicidal events.

## Methods

### Participants

Adolescents (N = 190) were enrolled from the greater New York City and Pittsburgh communities through psychiatric outpatient programs, emergency departments, medical center research registries, and social media. Participants included adolescents aged 13 to 18 years reporting a current affective and/or substance use disorder who owned a smartphone (Android, iPhone 7 or higher), and recruitment oversampled for current STB. Participants reporting an imminent risk of attempting suicide, active mania, current psychosis, or an autism spectrum diagnosis were excluded. Individuals were excluded from analysis if no geolocation data were ascertained (n = 4). The final sample (n = 186) included 43.5% (n = 81) reporting clinically meaningful suicidal ideation (ie, Scale for Suicide Ideation score ≥4) and 26.9% (n = 50) reporting ideation and a past-year suicide attempt (Table 1).

**Table 1.** Sociodemographic and Clinical History of Participants by Site

Characteristic	Participants, No. (%)			χ^2^ or *t*^a^	*P* value	Effect size
	All (N = 186)	New York (n = 84)	Pittsburgh (n = 102)			
Biological sex						
Male	38 (20.43)	13 (15.48)	25 (24.51)	1.79	.18	OR = 1.77
Female	146 (79.57)	71 (84.52)	77 (75.49)			
Age, mean (SD), y	16.43 (1.68)	16.44 (1.61)	16.42 (1.74)	*t* = 0.08	.94	*d* = −0.01
Race						
American Indian or Alaska Native	1 (0.54)	1 (1.19)	0 (0)	42.38	<.001	*V* = 0.21
Asian	19 (10.22)	14 (16.67)	5 (4.9)			
Black or African American	23 (12.37)	16 (19.05)	7 (6.86)			
White	106 (56.99)	27 (32.14)	79 (77.45)			
More than 1 race	21 (11.29)	12 (14.29)	9 (8.82)			
Unknown or not reported	16 (8.60)	14 (16.67)	2 (1.96)			
Hispanic ethnicity	32 (17.20)	26 (30.95)	6 (5.88)	18.60	<.001	OR = 0.14
Sexual/gender minority^b^	44 (53.01)	30 (56.6)	14 (46.67)	0.41	.52	OR = 0.67
Annual household income, $^c^						
<25 000	18 (11.69)	11 (17.19)	7 (7.78)	3.87	.14	*V* = 0.11
25 000-100 000	65 (42.21)	23 (35.94)	42 (46.67)			
≥100 000	71 (46.10)	30 (46.88)	41 (45.56)			
Current psychiatric disorders						
MDD	112 (60.22)	44 (52.38)	68 (66.67)	3.35	.07	OR = 1.81
Bipolar disorder	7 (3.76)	0 (0)	7 (6.86)	NA	NA	NA
Anxiety disorder^d^	167 (89.78)	73 (86.9)	94 (92.16)	0.87	.35	OR = 1.77
PTSD	28 (15.05)	11 (13.1)	17 (16.67)	0.22	.64	OR = 1.33
Substance use disorder	20 (10.75)	15 (17.86)	5 (4.9)	6.76	.009	OR = 0.24
Behavioral disorders^e^	66 (35.68)	28 (33.33)	38 (37.62)	0.20	.65	OR = 1.21
No. of current disorders, mean (SD)	2.85 (1.50)	2.55 (1.46)	3.10 (1.51)	*t* = 2.53	.01	*d* = 0.37
Baseline suicidal thoughts and behaviors, mean (SD)						
Past-week suicide ideation^f^	0.78 (1.87)	1 (1.95)	0.6 (1.79)	*t* = 1.45	.15	*d* = −0.22
Past-month suicide ideation^f^	4.14 (8.38)	5.65 (10.48)	2.89 (5.92)	*t* = −2.15	.03	*d* = −0.32
Past-month suicide plans^g^	0.32 (2.74)	0.7 (4.05)	0.01 (0.1)	*t* = −1.57	.12	*d* = −0.24
Past-year suicide attempts, No. (%)^h^	52 (27.96)	18 (21.43)	34 (33.33)	2.68	.1	OR = 1.83
Lifetime suicide attempts, No. (%)^h^	55 (29.57)	20 (23.81)	35 (34.31)	1.96	.16	OR = 1.67
Age of first attempt, y	14.71 (1.9)	14.45 (1.76)	14.86 (1.99)	*t* = 0.79	.44	*d* = 0.22

Abbreviations: *d*, Cohen *d*; MDD, major depressive disorder; NA, not applicable; OR, odds ratio; PTSD, current posttraumatic stress disorder; *V*, Cramer *V*.

^a^ Differences between New York and Pittsburgh participants are tested via *t* tests (for continuous variables) or χ^2^ tests (for categorical variables).

^b^ A baseline self-report sexual orientation item was added to the study protocol after the study was ongoing, such that 103 participants are missing this item. Sexual and gender minority reflects individuals who identify as nonheterosexual and/or with a gender identity differing from one’s sex assigned at birth.

^c^ Thirty-two participants are missing the annual household income item.

^d^ Anxiety disorders included current panic disorder, current agoraphobia, current separation anxiety disorder, current social anxiety disorder, current specific phobia, current generalized anxiety disorder.

^e^ Behavioral disorders included oppositional defiant disorder, conduct disorder, attention-deficit/hyperactivity disorder.

^f^ Past-week and past-month suicide ideation categories were measured in days.

^g^ Past-month suicide plan category was measured in days.

^h^ Past-year and lifetime suicide attempts were measured as percentage of sample.

### Procedure

The New York State Psychiatric Institute institutional review board approved study procedures, and data were collected from November 2018 through October 2023. Adolescent written assent and parental written consent were obtained for participants aged 13 to 17 years, while participants aged 18 years consented. At baseline, participants completed a comprehensive clinical battery, and they self-reported sociodemographic information, including race and ethnicity. Assessments were readministered at 1, 3, and 6 months; retention was excellent (1-month; n = 184 [98.9%]; 3-month: n = 183 [98.4%], 6-month: n = 181 [97.3%]). Participants also installed the Effortless Assessment Research System (EARS) app on their personal smartphones, which obtained passive sensor data related to mobility patterns and ESM data for 6 months.

### Clinical Assessment

#### Interviews

At baseline, participants were administered the Mini International Neuropsychiatric Interview for Children and Adolescents, Version 7.02 (MINI-KID^18^), a structured interview assessing *Diagnostic and Statistical Manual of Mental Disorders* (Fifth Edition) psychiatric disorders in adolescents. At each assessment, participants were administered the Self-Injurious Thoughts and Behaviors Interview (SITBI^19^) to assess the presence, frequency, and severity of self-injurious thoughts and behaviors. At baseline, adolescents reported on lifetime, past-year, past-month, and past-week STB. At follow-ups, participants reported on STB since last assessment. Participants also completed a structured interview at each assessment regarding psychiatric service utilization (see eMethods in Supplement 1 for reliability). The Columbia Suicide Severity Rating Scale (C-SSRS^20^) was administered to assess imminent STB risk (if indicated by clinical interviews or ESM). If imminent risk was detected, staff bridged participants to clinical services.

#### Self-Report Instruments

At baseline, participants completed the 21-item Scale for Suicidal Ideation (SSI^21^). Item scores ranged from 0 to 2, with higher scores reflecting greater past-week ideation severity. The last 2 items assessed attempts and were not included in analyses (α = 0.93).

### Smartphone Data

#### EARS Geolocation Data

EARS is a smartphone app for iOS and Android that collects passive sensor and ESM data.^22^ Although EARS collects a wide range of passive sensor data, hypotheses focused on GPS data (see eMethods in Supplement 1 for acquisition and security details). Raw GPS data were converted into daily estimates of: (1) entropy, (2) homestay, and (3) distance traveled. Entropy reflects the variability of time that participants spent at different locations, such that high entropy indicates participants spent closer to equal time across different locations, while low entropy indicates greater inequality in the time spent across locations. Homestay is the amount of time spent at one’s home (200-m radius). GPS coordinates of the home were estimated as the location where the participant spent the most time between 2 am and 6 am over the study period. Sensitivity analyses tested multiple home locations (eFigures 5, 7, and 8, eTable 5 in Supplement 1) and additional geolocation features (location variance, travel time; eTables 8 and 9 in Supplement 1). Distance traveled reflected the total daily distance (km) each participant traveled. Across participants, GPS data were available for 24 861 days (74.7%) out of an estimated 33 287 total days.

#### EARS Experience Sampling Methods

Each Wednesday at 9 am, participants received a prompt in EARS assessing: (1) suicidal ideation frequency, “In the past week, how often have you thought of killing yourself?,” rated on a scale from 1 (never) to 5 (all the time), (2) past-week suicide plan (yes/no), and (3) past-week suicide attempt (yes/no). Participants could respond from 9 am to 5 pm. If participants reported frequent suicidal ideation (≥4; “often” or “all the time”), a past-week suicide plan, and/or attempt, clinical staff contacted the participant to assess safety, and when necessary, bridge to emergency services (see eMethods in Supplement 1 for safety protocol).

#### Suicidal Outcomes

Suicidal events and ideation were examined separately. Suicidal events included attempts (ie, actual, aborted, and interrupted) as well as psychiatric hospitalizations and suicide-related emergency department visits as ascertained by follow-up SITBI assessments, weekly EARS surveys, and/or C-SSRS risk assessments. Dates for events were obtained using a timeline follow-back approach. The presence of clinically meaningful suicidal ideation was based on reporting past-week ideation (≥4) and/or suicide plans (yes) on weekly EARS surveys.

### Statistical Analysis

All analyses were completed in R version 4.3.0 (R Project for Statistical Computing), and a 2-sided *P* < .05 was considered statistically significant. Weekly mean entropy, homestay, and distance traveled (km) were computed on a participant level from each Wednesday to the following Tuesday, as the EARS weekly surveys were delivered every Wednesday. Weeks with less than 3 days of GPS data were excluded (N = 1150 weeks). Weekly geolocation metrics were decomposed into separate participant-mean-centered (ie, indicating weekly change from one’s mean) and participant-mean terms to parse within-participant vs between-participant associations.

#### Associations Between Geolocation Features and STB

Mixed-effects logistic regression models using the lme4 package^23^ tested whether geolocation features on a given week (Time_T-1_) were associated with suicidal outcomes during the following week (Time_T_). Within-participant and between-participant terms for weekly entropy, homestay, and distance traveled were included in the same model with a random intercept per participant (models with each geolocation feature separately yielded highly similar results; eTable 3 in Supplement 1). There were 3769 weeks included in analyses of suicidal events, and 1618 weeks in analyses of suicidal ideation (due to missing surveys; eFigure 3 in Supplement 1). All models covaried for weeks since baseline. Models also tested different temporal lags and potential bidirectional associations (ie, STB Time_T-1_ related to next-week geolocation Time_T_).

Between-person associations between mean geolocation metrics and suicidal events (as well as clinically meaningful suicidal ideation reported via ESM) were examined via the person-mean terms from the mixed-effects logistic regression models previously described. Linear regression also tested whether baseline suicidal ideation (SSI) was associated with between-participant differences in mean geolocation metrics.

#### Evaluating Accuracy

A leave-future-out validation approach was utilized to determine the accuracy with which geolocation predicted weeks when participants reported STB. Five mixed-effects logistic regression models were trained from the first 3-month half of the study period for each participant to test whether adding geolocation features improved STB prediction beyond models only using baseline features. Models included (1) all geolocation features (mean entropy, homestay, and distance traveled), (2) homestay only, (3) baseline features (SSI, site, sex assigned at birth, age, and telephone type), (4) homestay plus baseline features (features from models 2 and 3), and (5) all geolocation plus baseline features (features from models 1 and 3). Model outcomes were whether participants experienced suicidal events or ideation on a given week (Time_T_), and geolocation features were weekly (Time_T-1_) aggregates of entropy, homestay, and distance traveled. Next, model area under the receiver operating characteristic curve (AUC) was evaluated on the second 3-month half of each participants’ study period, and AUC uncertainty was calculated via bootstrapping (2000-fold; pROC::ci.auc R package).^24^ Random intercepts were not used in deriving risk estimates, such that accuracy was not influenced by individual differences in each outcome (eg, detecting risk of suicidal events based on prior suicidal events).

Geolocation features aggregated over the first month of the study were used to identify which participants were at risk for later STB. Separate logistic regression models assessed whether participants experienced suicidal events (n = 16) or weeks with suicidal ideation (n = 28) in the last 5 months of participation. Seventy percent (n = 129) of participants were randomly partitioned for a training model, and AUC was evaluated in the remaining 30% (n = 56) of held-out participants. This procedure was repeated 10 000 times with different seeds to derive uncertainty estimates for prediction performance.^25^

## Results

### Descriptive Analyses

A total of 186 participants were included in the study; 148 (79.57%) female; 19 (10.22%) Asian, 23 (12.37%) Black or African American, 106 (56.99%) White, 21 (11.29%) more than 1 race; 32 (17.20%) Hispanic; and mean (SD) age was 16.43 (1.68) years. A total of 33 suicidal events (8 actual attempts, 12 aborted attempts, 11 emergency department visits for suicidal concerns, and 7 hospitalizations for suicidal concerns; emergency department visits and hospitalizations were considered the same suicidal event as attempts if occurring within the prior 7 days) were reported across the study period by 20 unique participants (eFigure 4 in Supplement 1). Clinically meaningful suicidal ideation (inclusive of ideation ≥4 and “yes” to plans) was reported on 97 weekly surveys by 42 unique participants. Weekly STB ESM surveys were completed for 41% of weeks, and GPS data were collected on 75.8% of days during the study (see GPS distributions in eFigure 1 and 2 in Supplement 1). The likelihood of suicidal events did not significantly change as a function of time since baseline (*z* = −0.53; OR = 0.99 [95% CI, 0.94-1.03]; *P* = .60), although the occurrence of clinically meaningful ideation decreased over time (*z* = −2.25; OR, 0.96 [95% CI, 0.92-0.99]; *P* = .02).

### Associations Between Geolocation Features and STB

#### Week-by-Week Within-Person Analysis

**Figure 1.**
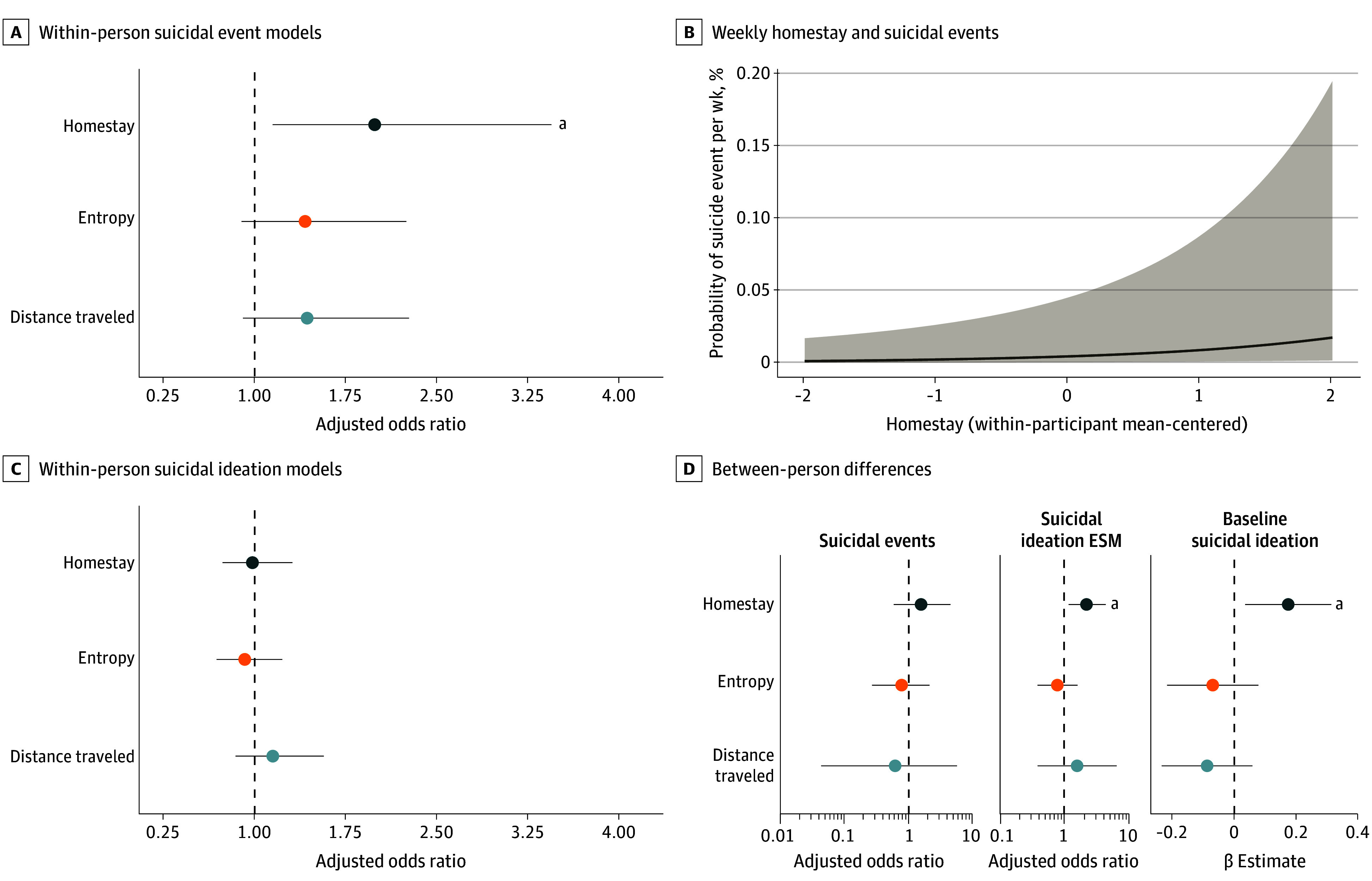
Within-Person and Between-Person Associations Between Geolocation Features and Suicidal Events A, Adjusted odds ratios and 95% CI for within-person associations between geolocation features and suicidal events the following week from mixed-effects models. All geolocation features were within-participant centered then standardized, such that odds ratios represent changes in odds of suicidal events in a given week given a 1 SD within-participant increase in the respective geolocation feature. B, Estimated probability of suicidal events (y-axis) as a function of differences in hours of homestay from a given participant’s mean (x-axis). The shaded gray area indicates 95% CI. Note: the y-axis is expressed as a percentage (all estimates indicate <1% likelihood of events). C, Adjusted odds ratios and 95% CI for within-person associations between geolocation features and suicidal ideation the next week. D, Between-person associations between geolocation features and suicidal thoughts and behaviors. The left 2 panes (suicidal events, suicidal ideation experience sampling methods [ESM]) show between-participants estimates from mixed-effects multilevel regression models predicting same-week outcomes. The right panel (baseline suicidal ideation) shows terms from separate linear regression models between baseline suicidal ideation and mean geolocation features. ^a^*P* < .05.

Greater homestay on a given week, relative to one’s own mean, was associated with a higher likelihood of suicidal events in the following week (Table 2). The odds of suicidal events were 1.99 (95% CI, 1.15-3.45) times as high in the subsequent week when homestay increased by 1 SD above one’s own mean (Figure 1A and 1B). Neither entropy nor distance traveled were associated with suicidal events. No geolocation features were significantly associated with next-week suicidal ideation (Figure 1C, Table 2). However, increased homestay was associated with greater odds of reporting clinically meaningful suicidal ideation during the same week (adjusted odds ratio [aOR], 1.47 [95% CI, 1.04-2.08]) (eFigure 6 in Supplement 1). Analyses considering varied covariates (including simpler models to minimize overfitting), multiple potential home locations, alternative coding of suicidal event dates, and missing data handling strategies indicated largely comparable results (eFigures 7-17, eTables 2, 3, 5, and 6 in Supplement 1).

**Table 2.** Associations Between Passive Geolocation Features With Next-Week Suicidal Events and Clinically Meaningful Ideation^a^

Outcomes and variables	β (SE)	aOR (95% CI)	*P* value
Suicidal events			
Intercept	−9.59 (1.207)	NA	NA
Homestay (within-participant)	0.69 (0.281)	1.99 (1.15-3.45)	.01
Entropy (within-participant)	0.35 (0.237)	1.42 (0.89-2.25)	.14
Travel distance (within-participant)	0.36 (0.236)	1.43 (0.90-2.28)	.13
Homestay (between-participant)	0.41 (0.645)	1.50 (0.42-5.31)	.53
Entropy (between-participant)	−0.22 (0.653)	0.80 (0.22-2.89)	.74
Travel distance (between-participant)	−0.01 (1.458)	0.99 (0.06-17.23)	.99
Weeks since baseline	−0.04 (0.030)	0.97 (0.91-1.02)	.24
Suicidal ideation (weekly experience sampling)			
Intercept	−4.56 (0.587)	NA	NA
Homestay (within-participant)	−0.02 (0.148)	0.98 (0.73-1.31)	.90
Entropy (within-participant)	−0.09 (0.149)	0.92 (0.69-1.23)	.56
Travel distance (within-participant)	0.14 (0.159)	1.15 (0.84-1.57)	.38
Homestay (between-participant)	0.57 (0.313)	1.77 (0.96-3.26)	.07
Entropy (between-participant)	−0.36 (0.341)	0.70 (0.36-1.37)	.30
Travel distance (between-participant)	0.21 (0.706)	1.23 (0.31-4.93)	.77
Weeks since baseline	−0.03 (0.020)	0.97 (0.93-1.01)	.15

Abbreviations: aOR, adjusted odds ratio; NA, not applicable.

^a^ Associations from mixed-effects logistic regression models. Site = Columbia University vs University of Pittsburgh; device = iOS vs Android; SSI = Scale for Suicidal Ideation; suicidal events = attempts, psychiatric hospitalizations, and/or emergency department visits for suicidal thoughts and behavior (STB) concerns; suicidal ideation: experience sampling STB prompts wherein suicidal ideation scores were ≥4 or suicide plans were “yes.” Homestay, entropy, and distance traveled (within) indicate person-mean-centered and *z*-scored variables, such that odds ratio reflected a 1 SD increase above one’s mean. Homestay, entropy, and distance traveled (between) indicate *z*-scored participant means, such that odds ratios reflect differences between a participant at the grand mean and a participant 1 SD greater than the grand mean. β estimates for regression coefficients and associated standard errors are expressed as log odds of the probability of respective outcomes. Results were similar for several sensitivity checks (eFigures 7-17 in Supplement 1).

**Figure 2.**
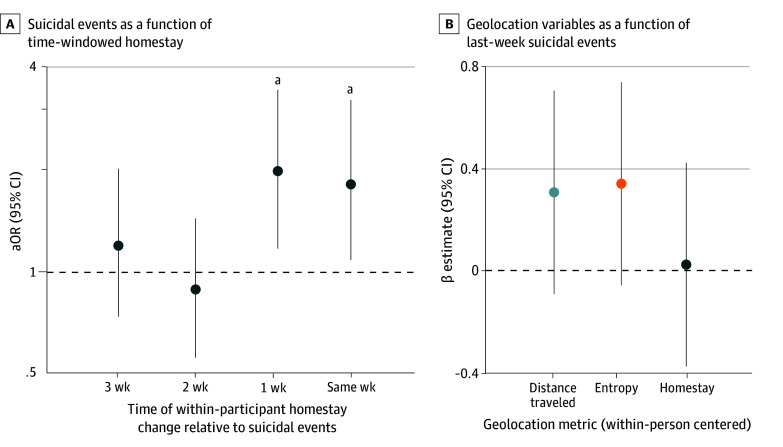
Within-Person Associations Between Geolocation Variables and Suicidal Events at Different Time Windows A, Odds ratios (y-axis) and 95% CI for within-person associations between homestay at different time windows and suicidal events, ranging from homestay aggregated for week Time_T-3_ to the same week. Significant positive associations between homestay and suicidal events were observed for week Time_T-1_ and the same week only. Estimates are shown from Bayesian mixed-effects logistic regression models, which were fit due to convergence issues with the frequentist Time _T-2_ model. For all time windows, Bayesian and frequentist models indicated highly similar results. B, β estimates and 95% CI for within-person associations between suicidal events (Time_T-1_) and geolocation variables the following week (Time_T_). No significant associations between suicidal events and geolocation variables the following week were observed. ^a^*P* < .05.

Models examining different temporal lags highlighted that the odds of suicidal events also were 1.80 (95% CI, 1.05-3.19) times higher during the same week when homestay increased 1 SD above one’s own mean. However, no significant associations were found for homestay 2 or 3 weeks prior (Time_T-2_ or Time_T-3_) to suicidal events (Figure 2A), and there were no significant associations between suicidal events (Time_T-1_) and geolocation variables the following week (Time_T_) (Figure 2B; eTables 1, 2, and 4 in Supplement 1).

#### Between-Person Analysis

There were no between-participant associations between geolocation variables and suicide events (Table 2). However, greater baseline suicidal ideation (SSI) associated with increased homestay (β = 0.17 [95% CI, 0.03-0.31]; *P* = .01). Participants with higher mean homestay were also more likely to report clinically meaningful suicidal ideation via ESM (aOR, 1.93 [95% CI, 1.01-3.67]; *P* = .045) (Figure 1D); this association was not significant using weekly geolocation features from Time_T-1_ (Table 2).

### Evaluating Accuracy for Suicidal Thoughts and Events

#### Weekly Performance

**Figure 3.**
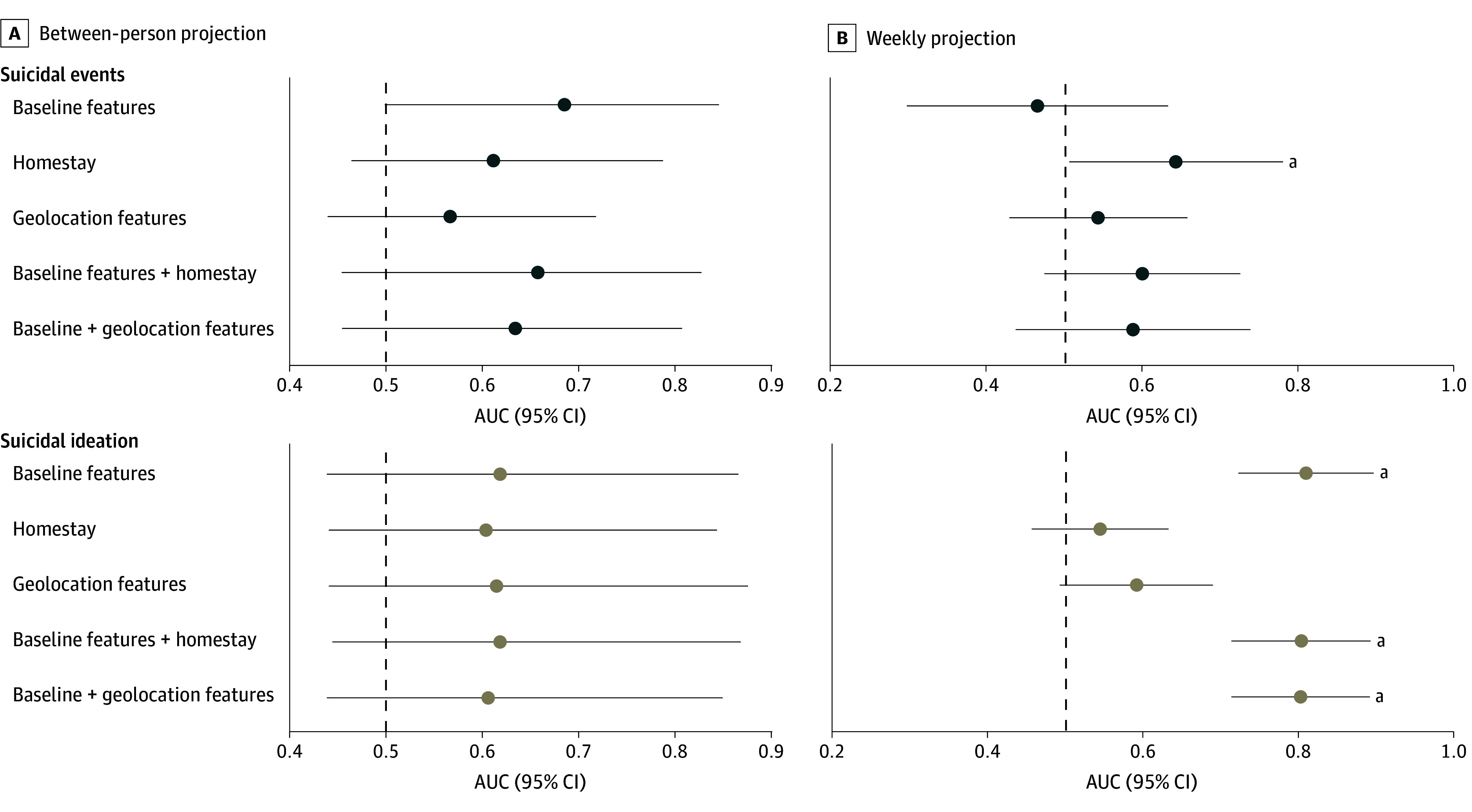
Accuracy of Models of Suicidal Ideation and Events A, Between-person identification of which participants will experience suicidal ideation (top) or events (bottom) during the last 5 months of the study window. The x-axis shows area under the receiver operating characteristics curve (AUC) scores and 95% CI within the held-out 30% of participants for logistic regression models using geolocation features (homestay, entropy, distance traveled) aggregated over the first month of the study window, only homestay, baseline features (baseline suicidal ideation, sex, site, age, device type), and the combination of geolocation and baseline features. B, Within-participant identification of which weeks participants will experience suicidal ideation (top) or events (bottom) using leave-future-out validation. The x-axis shows AUC scores and 95% CI within the held-out final half of the study (3 months) for each participant for models paralleling those in panel A, but with time-varying within-participant weekly geolocation features. As an external dataset was not available for validation analyses, model predictive performance metrics based on internal validation may be inflated. ^a^Above-chance performance (lower bound of 95% CI ≥0.5).

Model 2 including only homestay performed significantly above chance in detecting suicidal events on a weekly basis in leave-future-out validation, although accuracy was modest (AUC, 0.64 [95% CI, .50-0.78]) (Figure 3). No other model performed better than chance. Additionally, only models with baseline features detected weekly suicidal ideation above chance in held-out data; geolocation features did not improve performance (eTable 7 and eFigure 18 in Supplement 1).

#### Between-Person Performance

AUC scores in held-out participants indicated that geolocation features averaged over the first month did not improve the identification of which participants would experience suicidal events or ideation. No models identified which participants would experience either suicidal events or ideation above chance (Figure 3).

## Discussion

In the past 15 years, suicide rates among adolescents have increased by approximately 60%.^1^ Furthermore, 45% of individuals in the United States reside in communities with shortages of mental health professionals,^26,27^ and there is a critical need to develop scalable tools that detect suicide risk, especially tools that have the capacity to facilitate interventions when that risk is greatest. Acquiring passive sensor data through personal smartphones holds promise to monitor risk and to deliver timely interventions, as ownership of these devices is nearly ubiquitous.^8^ Our findings show that in a high-risk group, weekly increases in homestay—measured continuously via GPS data—were associated with the occurrence of next-week suicidal events. Although considerable work remains to improve the reliability, accuracy, and generalizability of these models, these findings offer a starting point for a new approach to suicide prevention research.

Increased homestay on a given week, but not entropy or distance traveled, was associated with a near 2-fold greater risk in the occurrence of suicidal events in the subsequent week. Analyses also corroborated the association between homestay and suicidal events in the same week, suggesting some temporal specificity in that homestay 2 or more weeks prior was not associated with suicidal events. Between-individual analyses highlighted greater average homestay among individuals reporting higher baseline suicidal ideation, and those reporting clinically meaningful ideation via ESM, relative to those who did not. Yet, there were no between-individual associations between geolocation features and suicidal events, highlighting potential differences in predictors for who may be at risk from when risk is greatest.

The findings are consistent with research linking greater homestay with depression,^11,12,13^ suggesting that increased isolation is related to symptom acuity. According to a 2023 United States Surgeon General report, we are amidst an epidemic of loneliness.^28^ Increasingly, youths are feeling disconnected from their communities, which for some, increases suicide risk. Tracking homestay via smartphones does not afford insight about what youths are doing in their homes but does signal that youths are withdrawing from activities outside the home. Among youths at risk for suicide, increased homestay may reflect a pattern of disconnecting from peers or activities (eg, sports, hobbies), or potentially, is the byproduct of feeling as though they are a burden to others in their lives.^29^ Understanding why increased homestay is an antecedent for suicide risk (perhaps through future ESM work assessing loneliness or disconnectedness) may provide important insights about how to intervene and what may be helpful to deploy during these periods of increased risk.

Although promising, the accuracy of our models was modest, which is unsurprising as modeling rare occurrences, such as suicidal events, can be difficult. There may be, however, opportunities to improve these models. Research focused on clinical indicators^30^ and natural language processing^31^ of electronic health records is taking strides in identifying who may be at risk for suicide. Integrating these modalities with mobile sensing may improve risk detection within specific clinical contexts (eg, following emergency department discharge). Additionally, our current approach focused on GPS data. The value of focusing on a specific sensor and associated features is that results are interpretable. However, EARS obtains a wide range of smartphone data that can be combined in more complex machine learning approaches to improve model accuracy. For example, sentiment in language derived from smartphone social communication predicts next-day mood^32^ and greater proportional use of personal pronouns predicts when youths are depressed,^33^ underscoring the clinical richness of these data. Integrating information across sensors may then capture a holistic portrait of patients, reflecting activity patterns, affective lability, and sleep, which together may better identify when individuals are at risk for STB.

### Limitations

This study has limitations. First, only 11% of participants reported a suicidal event, affecting the capacity to detect associations and reducing the generalizability. Second, as an independent dataset was not available for validation, performance metrics based on internal validation may have been inflated in our analyses, although most models did not perform above chance levels in predicting events. Third, the sample was primarily female and residing in the New York City or Pittsburgh areas. Thus, findings may not be entirely representative. Additionally, despite the promise of mobile sensor tracking, there are privacy concerns. Some families may not be comfortable providing geolocation data, affecting the scalability of this approach.

## Conclusions

In this case series study, the amount of time a participant spent at home was associated with increased risk of STB. Recent advancements in smartphone technology now afford unique opportunities to capture affective and behavioral dynamics that presage suicide risk; however, we remain in the early stages of actualizing this potential. Although this study’s approach had modest accuracy, it is one of the few scalable and unobtrusive measures of short-term suicide risk that has been demonstrated. Future work leveraging multimodal clinical and smartphone data may improve our understanding of when and how to intervene.

## Notes

Supplement 1. **eMethods.** Supplementary Materials **eTable 1.** Within-Person Associations Between Homestay and Suicide Events at Different Temporal Lags **eTable 2.** Within-Person Associations Between Homestay and Suicide Events at Different Temporal Lags Using the EARS+ Homestay Definition **eTable 3.** Associations Between Passive Geolocation Features With Next-Week Suicidal Events and Clinically Significant Ideation, Separate Models for Each Geolocation Feature **eTable 4.** Within-Person Associations Between Suicide Events and Geolocation Features the Following Week **eTable 5. **Associations From Multilevel Logistic Regression Models Using the EARS+ Homestay Definition **eTable 6.** Associations From Multilevel Logistic Regression Models Using a Recoding of the Timing of Suicidal Events (Sensitivity Analysis E) **eTable 7.** Evaluation of the Accuracy of Within-Participant Predictive Models of Suicidal Events and Ideation **eTable 8.** Exploration of Associations Between Additional Geolocation Features and Suicidal Events **eTable 9.** Exploration of Associations Between Additional Geolocation Features and Suicidal Ideation **eFigure 1.** Distributions of Participant-level Means of GPS Smartphone Metrics **eFigure 2.** Distributions for Weekly Aggregates of Participant-level Means of GPS Smartphone Metrics **eFigure 3.** Missingness of GPS Data and Weekly STB Surveys **eFigure 4.** Timing of Suicidal Events and Suicidal Ideation **eFigure 5.** Within-participant Correlations between Geolocation Measures **eFigure 6.** Within-person Associations between Homestay Variables and Suicidal Ideation at Different Time Windows **eFigure 7.** Within-person and Between-person Associations between Geolocation Features and Suicide Events **eFigure 8.** Associations between Geolocation Variables and Suicidal Events at Different Time Windows **eFigure 9.** Associations between Geolocation Variables and Suicidal Events the Following Week with Sensitivity Checks **eFigure 10.** Within-person and Between-person Associations between Geolocation Features and Suicidal Events Using the Recoded Timing **eFigure 11.** Associations between Geolocation Variables and Suicidal Events at Different Time Windows Using the Recoded Suicidal Event Timing Described in Sensitivity Check e **eFigure 12. **Multiverse Sensitivity Checks for Within-person Associations between Homestay and Next-week Suicidal Events **eFigure 13.** Multiverse Sensitivity Checks for Within-person Associations between Entropy and Next-week Suicidal Events under Different Covariate Scenarios **eFigure 14.** Multiverse Sensitivity Checks for Within-person Associations between Travel Distance and Next-week Suicidal Events under Different Covariate Scenarios **eFigure 15.** Multiverse Sensitivity Checks for Within-person Associations between Homestay and Next-week Suicidal Ideation under Different Covariate Scenarios **eFigure 16.** Multiverse Sensitivity Checks for Within-person Associations between Entropy and Next-week Suicidal Ideation under Different Covariate Scenarios **eFigure 17.** Multiverse Sensitivity Checks for Within-person Associations between Travel Distance and Next-week Suicidal Ideation under Different Covariate Scenarios **eFigure 18.** Evaluation of the Accuracy of Predictive Models of Suicidal Ideation and Events
eReferences

Supplement 2.
Data Sharing Statement
