# Supplementary material for: Using Smartphone GPS Data to Detect the Risk of Adolescent Suicidal Thoughts and Behaviors
Source: JAMA Netw Open. 2025 Jan 27;8(1):e2456429. doi: 10.1001/jamanetworkopen.2024.56429 (PMC11773992; doi:10.1001/jamanetworkopen.2024.56429)
Supplement: Supplement 1. — eMethods. Supplementary Materials eTable 1. Within-Person Associations Between Homestay and Suicide Events at Different Temporal Lags eTable 2. Within-Person Associations Between Homestay and Suicide Events at Different Temporal Lags Using the EARS+ Homestay Definition eTable 3. Associations Between Passive Geolocation Features With Next-Week Suicidal Events and Clinically Significant Ideation, Separate Models for Each Geolocation Feature eTable 4. Within-Person Associations Between Suicide Events and Geolocation Features the Following Week eTable 5. Associations From Multilevel Logistic Regression Models Using the EARS+ Homestay Definition eTable 6. Associations From Multilevel Logistic Regression Models Using a Recoding of the Timing of Suicidal Events (Sensitivity Analysis E) eTable 7. Evaluation of the Accuracy of Within-Participant Predictive Models of Suicidal Events and Ideation eTable 8. Exploration of Associations Between Additional Geolocation Features and Suicidal Events eTable 9. Exploration of Associations Between Additional Geolocation Features and Suicidal Ideation eFigure 1. Distributions of Participant-level Means of GPS Smartphone Metrics eFigure 2. Distributions for Weekly Aggregates of Participant-level Means of GPS Smartphone Metrics eFigure 3. Missingness of GPS Data and Weekly STB Surveys eFigure 4. Timing of Suicidal Events and Suicidal Ideation eFigure 5. Within-participant Correlations between Geolocation Measures eFigure 6. Within-person Associations between Homestay Variables and Suicidal Ideation at Different Time Windows eFigure 7. Within-person and Between-person Associations between Geolocation Features and Suicide Events eFigure 8. Associations between Geolocation Variables and Suicidal Events at Different Time Windows eFigure 9. Associations between Geolocation Variables and Suicidal Events the Following Week with Sensitivity Checks eFigure 10. Within-person and Between-person Associations between Geolocation Features and Suicidal E [file jamanetwopen-e2456429-s001.pdf]

Supplemental Online Content

Auerbach RP, Bloom PA, Pagliaccio D, et al. Using smartphone GPS data to improve the prediction of adolescent suicidal thoughts and behaviors. *JAMA Netw Open.* 2025;8(1):e2456429. doi:10.1001/jamanetworkopen.2024.56429

eTable 1. Within-Person Associations Between Homestay and Suicide Events at Different Temporal Lags

eTable 2. Within-Person Associations Between Homestay and Suicide Events at Different Temporal Lags Using the EARS+ Homestay Definition

eTable 3. Associations Between Passive Geolocation Features With Next-Week Suicidal Events and Clinically Significant Ideation, Separate Models for Each Geolocation Feature

eTable 4. Within-Person Associations Between Suicide Events and Geolocation Features the Following Week

eTable 5. Associations From Multilevel Logistic Regression Models Using the EARS+ Homestay Definition

eTable 6. Associations From Multilevel Logistic Regression Models Using a Recoding of the Timing of Suicidal Events (Sensitivity Analysis E)

eTable 7. Evaluation of the Accuracy of Within-Participant Predictive Models of Suicidal Events and Ideation

eTable 8. Exploration of Associations Between Additional Geolocation Features and Suicidal Events

eTable 9. Exploration of Associations Between Additional Geolocation Features and Suicidal Ideation

eFigure 1. Distributions of Participant-level Means of GPS Smartphone Metrics

eFigure 2. Distributions for Weekly Aggregates of Participant-level Means of GPS Smartphone Metrics

eFigure 3. Missingness of GPS Data and Weekly STB Surveys

eFigure 4. Timing of Suicidal Events and Suicidal Ideation

eFigure 5. Within-participant Correlations between Geolocation Measures

© 2025 Auerbach RP et al. *JAMA Network Open*.

eFigure 6. Within-person Associations between Homestay Variables and Suicidal Ideation at Different Time Windows

eFigure 7. Within-person and Between-person Associations between Geolocation Features and Suicide Events

eFigure 8. Associations between Geolocation Variables and Suicidal Events at Different Time Windows

eFigure 9. Associations between Geolocation Variables and Suicidal Events the Following Week with Sensitivity Checks

eFigure 10. Within-person and Between-person Associations between Geolocation Features and Suicidal Events Using the Recoded Timing

eFigure 11. Associations between Geolocation Variables and Suicidal Events at Different Time Windows Using the Recoded Suicidal Event Timing Described in Sensitivity Check e

eFigure 12. Multiverse Sensitivity Checks for Within-person Associations between Homestay and Next-week Suicidal Events

eFigure 13. Multiverse Sensitivity Checks for Within-person Associations between Entropy and Next-week Suicidal Events under Different Covariate Scenarios

eFigure 14. Multiverse Sensitivity Checks for Within-person Associations between Travel Distance and Next-week Suicidal Events under Different Covariate Scenarios

eFigure 15. Multiverse Sensitivity Checks for Within-person Associations between Homestay and Next-week Suicidal Ideation under Different Covariate Scenarios

eFigure 16. Multiverse Sensitivity Checks for Within-person Associations between Entropy and Next-week Suicidal Ideation under Different Covariate Scenarios

eFigure 17. Multiverse Sensitivity Checks for Within-person Associations between Travel Distance and Next-week Suicidal Ideation under Different Covariate Scenarios

eFigure 18. Evaluation of the Accuracy of Predictive Models of Suicidal Ideation and Events

eReferences

This supplemental material has been provided by the authors to give readers additional information about their work.

eMethods. Supplementary Materials

Clinical Interviews. All study staff received ~50 hours of training prior to conducting clinical interviews. Supervised training included didactics, role playing, and direct observation, and weekly case conferences throughout the study. Staff only conducted interviews after receiving certification, and there were annual re-certifications to prevent clinical drift. To assess the inter-rater reliability in our sample, 10% of baseline interviews were randomly selected for both the Mini International Neuropsychiatric Interview for Children and Adolescents, Version 7.02 (MINI-KID[1]) and Self-Injurious Thoughts and Behaviors Interview (SITBI[2]). The inter-rater reliability for the MINI-KID[1] was good (κ=0.73) and was excellent for the SITBI[2] (κ=0.86).

EARS Geolocation Data. GPS metrics relied on the acquisition of latitude and longitude location coordinates. Whereas iOS smartphones collect data when there is ≥100 meters of movement, Android-based smartphones obtained GPS sensor location data every 15 minutes. This sampling rate collects sufficient data to make inferences about mobility patterns while, at the same time, minimizing smartphone battery drainage. Latitude and longitude GPS data were then converted into a mobility trace defined by a sequence of flights and pauses. Flights are defined as segments of linear movement and pauses are periods when a person is stationary. The curved movement was approximated by multiple sequential flights. If a missing interval was flanked by two pauses at the same location (i.e., within 50 meters), the missing interval was assumed to be a longer pause at the same location. As the data stream has long periods of structured missingness, a resampling method was used to estimate a connected path of flights and pauses. Each missing period was filled with random draws from the participants' empirical

© 2025 Auerbach RP et al. *JAMA Network Open*.

distributions of observed flights and pauses to create complete paths that reflect individuals' observed mobility patterns.

EARS Data Security. Data collected through EARS is encrypted prior to being uploaded to the HIPAA-compliant cloud computing service, and only the research team has the capacity to download and decrypt it. The encryption protocol for data collected using EARS was as follows. After the data sensors generated the data, data were then transmitted to Azure. When transmitting the data to Azure, EARS used a Secure Socket Layer (SSL) connection to the server, meaning all data in transit are encrypted a second time using the industry standard for encrypting data travelling between networks. After transmission to the cloud, the EARS apps then deleted the unencrypted data from the phone's memory. Upon upload to Amazon Web Server, the data are then protected by Microsoft's Server-Side encryption, which used 256-bit AES encryption. By encrypting the user data during transmission and storage, we ensured the data cannot be accessed at any time by anyone outside our team. Upon completion of or withdrawal from the study, a participant's uninstallation of the EARS apps tool automatically deleted all encrypted EARS data residing on the phone. To access the data, research staff downloaded data directly from the Amazon Web Server. Data included a de-identified random alphanumeric ID to connect with other study data.

Safety Protocol. If a participant reported a score of ≥4 (scale of 1-5) on the weekly suicidal ideation prompt and/or "Yes" to reporting a past week suicidal plan or attempt, this triggered a textbox on an adolescent's smartphone directing the adolescent to clinical resources, "*Based on your response, you indicated that you may feel unsafe. We encourage you to talk with your parent or guardian as soon as possible. If you cannot keep yourself safe, please call 911 or visit the closest emergency department. There also are hotlines that may be of help to you: (a) 1-*

© 2025 Auerbach RP et al. *JAMA Network Open*.

800-SUICIDE, (b) 1-888-SUICIDE, (c) 1-800-273-TALK, (d) 1-800-252-TEEN (Hotline for Teens), (e) 1-800-850- 8078 (Hotline for LGBT Teens)." Additionally, these responses also initiated a text message and email to all clinical staff. Upon receipt of the alert, the designated on-call clinical staff then contacted the adolescent participant. Once reached by telephone, clinical staff administered the Columbia Suicide Severity Rating Scale to assess imminent suicide risk.[3] If necessary, clinical staff developed safety plans and bridged to emergency clinical services. In cases of all minors and when permitted by participants 18 years or older, clinical staff then discussed the assessment and associated action plan with parents.

Data Processing and Analytic Overview

EARS. Prior to generating geolocation metrics for entropy, homestay, and average distance traveled, several data processing steps were completed, removing days that included: (i) fewer than 5 geolocation captures (N=1,525 days), (ii) travel distances greater than 1,000 km (N=268 days), (iii) homestay and travel time surpassing 24 hours (N=2,344 days), and (iv) homestay days greater than 23 hours when the distance from home was greater than 50 km (N=10 days). Further, to reduce the potential impact of outliers, travel distances and entropy were winsorized using a within-subject approach (winsorized to ±1.5x the interquartile range for each participant).

Additional Geolocation Features. We conducted follow-up analyses of two additional geolocation features, location variance and travel time. Location variance was calculated as the variance in the coordinates of latitude and longitude coordinates visited, or $\log(sd(longitude)^2 + sd(latitude)^2)$ over the course of a 24-hour period. Travel time was calculated as the time spent in transit (or between stop locations) over the course of a 24-hour period. As with the geolocation

© 2025 Auerbach RP et al. *JAMA Network Open*.

features in the main text, we created within-person centered and person-mean terms for these two features to parse within-participant from between-participant associations.

Sensitivity Checks: Several sensitivity checks for explanatory analyses were conducted. All explanatory analyses were re-run using multiple possible home location calculations (a), and sensitivity checks b-f were conducted for explanatory analyses of associations between geolocation variables and suicidal events the following week.

a) *Using Multiple Possible Home Location Calculations (EARS+).* Inspection of the EARS data indicated that some participants were spending little time at the home location calculated via the EARS algorithm (e.g., N=13 participants with 0 hours at home for ≥50% of days). To more broadly capture times spent at multiple potential home locations (especially for participants who may have moved homes, moved to college, or alternated staying at homes of different parents/guardians), we created a homestay variable where a participant was considered home when at: (a) the location that the participant spent the most time between 2:00-6:00AM over the study period, (b) the location that the participant spent the most time between 2:00AM-6:00AM over the first 14 days of the study period, and (c) the location of the participant's mailing address as reported at the baseline visit. This more inclusive homestay variable is referred to as the "EARS+" homestay calculation, while the default EARS homestay calculation is referred to as the "EARS" homestay calculation. Supplemental descriptive and explanatory analysis results compare findings using this EARS+ homestay variable to the one derived from the original EARS home location. Primary analyses reported in the main manuscript report results using the default EARS homestay calculation to maximize generalizability to potential clinical applications (e.g., where timely ascertainment of multiple home

© 2025 Auerbach RP et al. *JAMA Network Open*.

locations may not be possible). As entropy and distance traveled did not rely on a calculation of participants' home locations, these sensitivity analyses were not necessary for these geolocation measures.

b) *Using Joint Analysis and Imputation of Data via Bayesian Models.* To conduct analyses robust to the possibility of data missing at random (MAR), we conducted sensitivity checks using a Bayesian imputation strategy. Using the JoinAI R package,[4] we fit analysis models paralleling the formulae of those reported in the main manuscript using joint analysis and imputation (at a weekly level) of incomplete variables. Thus, analysis and imputation were conducted simultaneously within a fully Bayesian framework. Missing values for geolocation features were modeled using linear mixed models ('lmm') as a function of all other covariates, while missing suicidal event outcomes were modeled via logistic mixed models ('glmm_logit') as a function of all other covariates. Package default priors were used, and all models were fit using 2000 iterations. For Bayesian analyses, whether the 95% credible interval (CI) excluded 0 was taken as a proxy for significance.

c) *Covariates.* Multiverse-style sensitivity analyses were conducted to examine robustness of primary results to inclusion of several covariates with potential effects on geolocation patterns and STB. Covariates were (i) Summer break: a binary covariate for summer breaks from school. This covariate was coded as 1 for weeks falling within July or August and 0 for other times of year, (ii) School closure: to account for potential COVID-19 confounds, models also included a binary variable indicating weeks when local public high schools were closed due to the pandemic, (iii) Baseline suicidal ideation as reported via the Beck Scale for Suicidal Ideation, (iv), Site (New York vs. Pittsburgh), (v) sex

© 2025 Auerbach RP et al. *JAMA Network Open*.

assigned at birth, (vi), age, (vii) phone type (Android vs. iOS), and geolocation features aside from the focal predictor. Multiverse analyses are a method of running all possible combinations of reasonable analyses to examine the robustness of results.[5] A total of 12 covariates were tested, for a total of 4096 (e.g. 2^{12}) possible model specifications for each multiverse. A specific set of minimal models containing predictors for each geolocation feature separately (e.g. both the within-participant and between-participant homestay term, but excluding other geolocation features) are also displayed in **eTable 3**.

d) *Examining Associations of Each Geolocation Predictor Separately.* As geolocation features were somewhat correlated with one another (**eFigure 5**), follow-up analyses also examined associations between each predictor and suicidal events in the following week without inclusion of any other geolocation features in the model. These models were each pared down to only 3 predictors (plus random intercepts) to minimize potential for overfitting and included solely the respective within-participant and between-participant features, plus a predictor for time since baseline.

e) *Coding of Suicidal Event Timing.* One participant reported a suicide attempt for which they could not recall the exact date, which was by default coded as occurring on the first day of the month in primary analyses. However, as the participant also reported a suicide-related ED visit on the fourth day of that month with higher certainty, it may have been that this suicide attempt occurred on the fourth day of that month as well. In this case, the first and fourth days of the month fell into different consecutive Weds-Tues week periods. Although primary analyses were run considering these reports as suicidal events in two consecutive weeks, sensitivity analyses were also run considering these reports as occurring on the same day (the fourth of the month) within a single week.

© 2025 Auerbach RP et al. *JAMA Network Open*.

f) *Geolocation Associations with Suicidal Events Covarying for Suicidal Ideation.* To understand whether geolocation features predicted next-week suicidal events above and beyond suicidal ideation, we ran an additional model for suicidal events parallel to primary analyses (**Table 2**) with an added binary covariate for weekly suicidal ideation (at Time_{T-1}) reported via EARS ESM.

Heterogeneity of Within-Participant Associations Between Geolocation & Suicidal Events. To test whether between-participant differences in geolocation features accounted for heterogeneity in within-participant associations, we tested a set of models (parallel to those reported in the main text in **Table 2**) with interaction terms between the within-person and respective between-person geolocation term (e.g. Homestay [within] X Homestay [between]). We also tested a follow-up model adding a random slope for each participant for the within-participant Homestay term to examine the heterogeneity of this association across the sample. This mixed-effects logistic regression with random slopes was fit using Bayesian estimation with the brms R package.[6] Beta estimates and their associated standard deviations are reported in log odds.

Validation of a Model Including only a Baseline Suicidal Ideation Feature. In the validation analyses, the Homestay only model was the only model to perform above chance in predicting suicidal events on a weekly basis. To rule out the potential explanation that this model was simply overfitting to a lesser degree than other models because it had the fewest predictors, we also validated an additional model no more complex than the Homestay model with only baseline suicidal ideation (1 predictor + random intercepts) as a feature.

© 2025 Auerbach RP et al. *JAMA Network Open*.

Results

Descriptive

Distributions of Geolocation Features. Distributions of participant-level mean (**eFigure 1)** and within-participant centered (**eFigure 2)** geolocation features are shown. Compared to the default EARS algorithm's estimate (M_{EARS+}=13.03 hours, SD=4.90), including multiple potential home locations in the calculation of homestay ("EARS+", M_{EARS+}=14.70 hours, SD=3.91) significantly increased the estimated average time spent at home (t(180)=7.55, $p<.001$; **eFigure 1B**). However, distributions of within-participant mean-centered weekly homestay estimates were highly similar between EARS and EARS+ calculations (**eFigure 2B)**.

Missing Data. Temporal distributions of missing GPS and weekly STB survey data are shown in **eFigure 3.**

Temporal Distribution of STB. Timing of weeks with suicide events or clinically significant suicidal ideation relative to baseline visits is shown in **eFigure 4**.

Correlations Between Geolocation Features. Within-participants, weekly travel distance was negatively associated with homestay for most participants (**eFigure 5)**. Correlations between other pairs of geolocation features tended to be weaker. Weekly homestay using the default EARS calculation was strongly correlated with homestay when the EARS+ calculation was used.

Associations Between Baseline Self-Reported Suicidal Ideation and STB During the Study Window. Baseline suicidal ideation reported via the Scale for Suicidal Ideation (SSI) was associated with higher odds of clinically significant suicidal ideation reported via weekly ESM (aOR=3.40, 95% CI [2.25, 5.13]). However, self-reported baseline suicidal ideation was not associated with the odds of suicidal events (aOR=1.78, 95% CI [0.83, 3.83]).

© 2025 Auerbach RP et al. *JAMA Network Open*.

Associations between Geolocation Features and STB

Temporal Specificity of Homestay and Suicide Events. Statistics for within-participant associations between homestay and suicide events at different temporal lags are shown in **eTables 1 and 2.** Statistics for associations between suicide events and geolocation features the next week are shown in **eTable 4.**

Temporal Specificity of Associations Between Geolocation Features and Suicidal Ideation. Although there was a significant concurrent association between same-week homestay and weekly suicidal ideation reported via ESM, no significant associations were found between homestay 1, 2 or 3 weeks prior ($Time_{T-2}$ or $Time_{T-3}$) **(Table 2, eFigure 6).** Further, no significant associations were found between weekly suicidal ideation and geolocation features the next week. No significant within-participants associations between entropy or travel distance with weekly suicidal ideation were found at any time window.

Additional Geolocation Features. Follow-up analyses examined associations between two additional geolocation features, location variance and travel time, indicated no significant within-person or between-person associations with suicidal events or suicidal ideation **(eTables 8 and 9)**.

Sensitivity Checks:

(a) Results remained very similar using the EARS+ homestay calculation (which included multiple home locations) compared to the default EARS homestay calculation, such that all decisions based on the p<.05 threshold for significance remained the same. **eFigures 7-8** parallel main manuscript **Figures 1-2,** and **eTable 5** parallels main manuscript **Table 2** using the EARS+ homestay calculation.

© 2025 Auerbach RP et al. *JAMA Network Open*.

(*b*) Results remained similar for all other sensitivity checks for associations between geolocation features and suicidal events the using joint analysis and imputation of missing values. A further sensitivity check of the between-participants association between average homestay and suicidal ideation reported via ESM using joint analysis and imputation with the EARS+ homestay calculation indicated a similar significant positive association to the primary analyses (aOR=1.55, 95% CI [1.08, 2.19]).

(*c*) Multiverse sensitivity checks to 4096 possible combinations of covariates yielded results highly consistent with those reported in the primary analyses. Concurrent with the analysis in the main manuscript, most (93.4%) of sensitivity checks indicated a significant positive association between homestay and odds of a suicidal event the following week (**eFigure 12)**. Multiverse analyses did not identify any significant relationships between distance traveled or entropy and suicidal events the following week, or significant relationships between any geolocation features and suicidal ideation the following week (**eFigures 13-17)**.

(*d*) A set of models examining each geolocation feature separately (e.g. to minimize potential for overfitting) yielded highly similar results to those reported in the primary analyses (**eTable 3)**.

(e) Models using an alternative coding of the timing of one suicidal event indicated highly similar results to those reported in the primary analyses **(eTable 5, eFigures 10-11)**.

(f) A sensitivity check model with an added binary covariate for weekly suicidal ideation reported via EARS indicated similar results to the primary analyses, with a significant within-person association between homestay and next-week suicidal events (aOR=3.55, 95% CI [1.16, 10.83], p=.026).

© 2025 Auerbach RP et al. *JAMA Network Open*.

Heterogeneity of Within-Participant Associations Between Geolocation Features and Suicidal Events. Analyses revealed no significant within X between-participant term interactions on next-week suicidal events for the homestay (β=-.01, 95% CI [-.70, .69]), entropy (β=-.08, 95% CI [-.68, .52]), or travel distance features (β=.20, 95% CI [-1.15, 1.55]). However, a model including random slopes indicated significant between-participant heterogeneity in the within-participant association between homestay and next-week suicidal events, such that the heterogeneity was over half the magnitude of the fixed-effect estimate (SD(β)=.47, 95% CI [.02, 1.14], relative to β=0.85, 95% CI [-.13, 2.06]). When including random slopes, the fixed within-participant effect for homestay on next-week suicidal events was not significant, although we note this is likely because the majority of participants experienced no suicidal events during the study period.

Evaluating Predictive Accuracy for Suicidal Thoughts and Events

Weekly Prediction of STB. Model performance metrics for leave-future-out validation are shown in **eTable 7.** Base rates for the occurrence of STB in a given week across the entire dataset (0.054 for suicidal ideation, 0.007 for suicide events) were used to threshold binary model predictions to calculate sensitivity and specificity. For many models, sensitivity was 0 and specific was 0 because zero instances of STB outcomes were predicted, highlighting the overarching challenge of predicting rare outcomes in suicide research more broadly.

In addition, a simple model with only Baseline suicidal ideation as a feature performed similarly to the Baseline features model in both prediction of suicidal events (e.g. not predicting events above chance) and suicidal ideation (**eFigure 18)**. As this model had the same number of predictors as the Homestay only model, this provides evidence against the account that the Homestay only model performed best simply due to being less complex than other models.

© 2025 Auerbach RP et al. *JAMA Network Open*.

eTable 1. Within-person Associations between Homestay and Suicide Events at Different Temporal Lags

Timing	aOR	95% CI
Same Week	1.80	1.05, 3.19
T - 1 Week	1.96	1.18, 3.41
T - 2 Weeks	0.89	0.56, 1.41
T - 3 Weeks	1.19	0.73, 1.97

Note. Within-person associations between homestay and suicide events at different temporal lags from Bayesian multilevel logistic regression models (as plotted in main manuscript **Fig 2A)**. Bayesian models were fit due to convergence issues with the frequentist $Time_{T\text{-}2}$ model. For all time windows, Bayesian and frequentist models indicated highly similar results. aOR=odds ratio adjusted for covariates. Temporal lags indicate timing of homestay relative to suicide events (e.g. T-1 Week indicates homestay measured in the 7-day period prior to suicide events, T-2 weeks indicates homestay measured in the 7-day period 2 weeks prior to suicide events).

© 2025 Auerbach RP et al. *JAMA Network Open*.

eTable 2. Within-person Associations between Homestay and Suicide Events at Different Temporal Lags using the EARS+ Homestay definition

Timing	aOR	95% CI
Same Week	1.81	1.07, 3.2
T - 1 Week	1.80	1.07, 3.12
T - 2 Weeks	0.90	0.58, 1.43
T - 3 Weeks	1.13	0.69, 1.92

Note. Within-person associations between homestay and suicide events at different temporal lags from Bayesian multilevel logistic regression models using the EARS+ Homestay definition incorporating multiple potential home locations (parallel to main manuscript **Fig 2A)**. Bayesian models were fit due to convergence issues with the frequentist Time $_{T-2}$ model. For all time windows, Bayesian and frequentist models indicated highly similar results. aOR=odds ratio adjusted for covariates. Temporal lags indicate timing of homestay relative to suicide events (e.g. T-1 Week indicates homestay measured in the 7-day period prior to suicide events, T-2 weeks indicates homestay measured in the 7-day period 2 weeks prior to suicide events).

© 2025 Auerbach RP et al. *JAMA Network Open*.

eTable 3. Associations between Passive Geolocation Features with Next-Week Suicidal Events and Clinically Significant Ideation, Separate Models for Each Geolocation Feature

		Entropy Model			Homestay Model			Travel Distance Model		
**Outcomes**	**Variables**	**OR**	**95% CI**	**p**	**OR**	**95% CI**	**p**	**OR**	**95% CI**	**p**
**Suicidal Events**	Intercept	---	---	---	---	---	---	---	---	---
	Entropy (within)	1.36	0.88, 2.10	0.166						
	Entropy (between)	0.21	0.00, 145.03	0.643						
	Homestay (within)				1.61	1.01, 2.57	**0.045**			
	Homestay (between)				1.08	0.85, 1.39	0.523			
	Travel Distance (within)							1.21	0.81, 1.81	0.359
	Travel Distance (Between)							0.99	0.93, 1.06	0.819
	Weeks Since Baseline	0.98	0.92, 1.03	0.401	0.97	0.91, 1.03	0.276	0.98	0.92, 1.03	0.392
**Suicidal Ideation (Weekly Experience Sampling**	Intercept	---	---	---	---	---	---	---	---	---
	Entropy (within)	0.95	0.72, 1.26	0.712						
	Entropy (between)	0.08	0.00, 2.37	0.145						
	Homestay (within)				0.94	0.72, 1.24	0.676			
	Homestay (between)				1.14	1.01, 1.28	**0.036**			
	Travel Distance (within)							1.14	0.85, 1.53	0.37
	Travel Distance (Between)							0.99	0.96, 1.02	0.544
	Weeks Since Baseline	0.97	0.93, 1.01	0.144	0.97	0.93, 1.01	0.146	0.97	0.93, 1.01	0.149

Note. Associations from separate mixed-effects logistic regression models (e.g. one model for each of Entropy, Homestay, and Travel Distance for each outcome). SE=Standard Error; aOR=odds ratio adjusted for covariates; Suicidal Events= attempts, psychiatric hospitalizations, and/or emergency department visits for STB concerns; Suicidal Ideation: Experience sampling STB prompts wherein suicidal ideation scores were ≥4 or suicide plans were 'yes'. Homestay, entropy, and distance traveled (within) indicate person-mean-centered and z-scored variables, such that OR reflected a 1SD increase above one's mean. Homestay, entropy, and distance traveled (between) indicate z-scored participant means, such that ORs reflect differences between a participant at the grand mean and a participant 1SD > the grand mean. All models included a covariate for the number of weeks since the baseline visit. *p*-values<.05 are bolded.

© 2025 Auerbach RP et al. *JAMA Network Open*.

eTable 4. Within-person Associations between Suicide Events and Geolocation Features the Following Week

Variable	Beta	SE	95% CI	p
Homestay	0.02	0.203	-0.38, 0.42	0.91
Entropy	0.34	0.203	-0.06, 0.74	0.09
Distance Traveled	0.31	0.203	-0.09, 0.70	0.13

Note. Within-person associations between suicide events and geolocation features the following week from multilevel linear regression models (as plotted in main manuscript **Fig 2B)**. Standardized beta estimates are shown in the second column. No associations were significant.

© 2025 Auerbach RP et al. *JAMA Network Open*.

eTable 5. Associations from multilevel logistic regression models using the EARS+ homestay definition

Outcomes	Variables	Beta	SE	aOR	95% CI	*p*
**Suicide Events**	Intercept	-9.75	1.214	---	---	---
	Homestay (within)	0.61	0.283	1.83	1.05, 3.19	**0.032**
	Entropy (within)	0.26	0.235	1.3	0.82, 2.06	0.263
	Travel Distance (within)	0.36	0.241	1.43	0.89, 2.29	0.14
	Homestay (between)	0.04	0.656	1.04	0.29, 3.78	0.947
	Entropy (between)	-0.18	0.701	0.83	0.21, 3.29	0.794
	Travel Distance (between)	-0.03	0.682	0.97	0.25, 3.69	0.964
	Weeks Since Baseline	-0.04	0.03	0.97	0.91, 1.02	0.247
**Suicidal Ideation (Weekly Experience Sampling)**	Intercept	-4.85	0.678	---	---	---
	Homestay (within)	-0.07	0.152	0.93	0.69, 1.25	0.64
	Entropy (within)	-0.01	0.151	0.99	0.74, 1.33	0.941
	Travel Distance (within)	0.14	0.164	1.15	0.83, 1.58	0.392
	Homestay (between)	0.54	0.354	1.72	0.86, 3.44	0.126
	Entropy (between)	-0.35	0.359	0.7	0.35, 1.42	0.324
	Travel Distance (between)	0.17	0.329	1.18	0.62, 2.25	0.616
	Weeks Since Baseline	-0.03	0.021	0.97	0.93, 1.01	0.17

Note. Associations from multilevel logistic regression models using the EARS+ homestay calculation including multiple possible home locations (parallel to main manuscript **Table 2**). SE=Standard Error; aOR=odds ratio adjusted for covariates, Site=Columbia University vs. University of Pittsburgh; Device=iOS vs. Android; SSI=Scale for Suicidal Ideation; Suicide Events= attempts, psychiatric hospitalizations, and/or emergency department visits for STB concerns; Suicidal Ideation: Experience sampling STB prompts wherein suicidal ideation scores were ≥4 or suicide plans were 'yes'. Homestay, entropy, and distance traveled indicated person-mean-centered and z-scored variables, such that OR reflected a 1SD increase above one's mean. Beta estimates for regression coefficients and associated standard errors are expressed as log odds of the probability of respective outcomes. *p*-values<.05 are bolded.

© 2025 Auerbach RP et al. *JAMA Network Open*.

eTable 6. Associations from multilevel logistic regression models using a recoding of the timing of suicidal events (sensitivity analysis e)

Variables	Beta	SE	aOR	95% CI	*p*
Intercept	-9.43	1.213	---	---	---
Homestay (within)	0.62	0.278	1.85	1.08, 3.2	**0.026**
Entropy (within)	0.32	0.237	1.38	0.86, 2.19	0.179
Travel Distance (within)	0.32	0.238	1.37	0.86, 2.19	0.184
Homestay (between)	0.40	0.64	1.49	0.43, 5.22	0.532
Entropy (between)	-0.21	0.647	0.81	0.23, 2.89	0.751
Travel Distance (between)	-0.03	1.451	0.97	0.06, 16.69	0.984
Weeks Since Baseline	-0.04	0.030	0.96	0.90, 1.02	0.178

Note. Associations from multilevel logistic regression models using a recoding of the timing of suicidal events (parallel to main manuscript **Table 2**). SE=Standard Error; aOR=odds ratio adjusted for covariates, Suicide Events= attempts, psychiatric hospitalizations, and/or emergency department visits for STB concerns. Homestay, entropy, and distance traveled indicated person-mean-centered and z-scored variables, such that OR reflected a 1SD increase above one's mean. Beta estimates for regression coefficients and associated standard errors are expressed as log odds of the probability of respective outcomes. *p*-values<.05 are bolded.

© 2025 Auerbach RP et al. *JAMA Network Open*.

eTable 7. Evaluation of the Accuracy of Within-participant Predictive Models of Suicidal Events and Ideation

Outcome	Model	AUC [95% CI]	Sensitivity	Specificity
Suicidal Events	Baseline + Geolocation Features	0.59, [0.44, 0.74]	0	1
	Baseline Features	0.46, [0.30, 0.63]	0	1
	Baseline Features + Homestay	0.60, [0.47, 0.72]	0	1
	Baseline SI Only	0.55, [0.38, 0.72]	0	1
	Geolocation	0.54, [0.43, 0.66]	0	1
	Homestay	**0.64, [0.50, 0.78]**	0	1
Suicidal Ideation	Baseline + Geolocation Features	**0.80, [0.71, 0.89]**	0.54	0.88
	Baseline Features	**0.81, [0.72, 0.90]**	0.63	0.89
	Baseline Features + Homestay	**0.80, [0.71, 0.89]**	0.63	0.89
	Baseline SI Only	**0.79, [0.71, 0.87]**	0.31	0.89
	Geolocation	0.59, [0.49, 0.69]	0	1
	Homestay	0.54, [0.46, 0.63]	0	1

Note. Evaluation of the accuracy of within-participant predictive models of suicidal events (top) and ideation (bottom). Within-participant prediction was conducted using leave-future-out validation. AUC, sensitivity, and specificity metrics are shown within the held-out final half of the study (3-months). Above-chance AUC metrics (lower bound of 95% CI >= 0.5) are bolded.

© 2025 Auerbach RP et al. *JAMA Network Open*.

eTable 8: Exploration of Associations Between Additional Geolocation Features and Suicidal Events

	Time T			Time T			Time T-1			Time T-1		
Predictors	*Odds Ratios*	*95% CI*	*p*	*Odds Ratios*	*95% CI*	*p*	*Odds Ratios*	*95% CI*	*p*	*Odds Ratios*	*95% CI*	*p*
Intercept	---	---	---	---	---	---	---	---	---	---	---	---
Location Variance (Within)	1.01	0.65 – 1.57	0.980				1.36	0.88 – 2.10	0.166			
Location Variance (Between)	0.95	0.22 – 4.01	0.941				0.72	0.21 – 2.47	0.605			
Travel Time (Within)				1.11	0.71 – 1.74	0.642				1.28	0.86 – 1.90	0.228
Travel Time (Between)				0.46	0.07 – 3.17	0.430				0.45	0.06 – 3.22	0.426
Weeks Since Baseline	0.98	0.92 – 1.03	0.410	0.97	0.92 – 1.03	0.305	0.98	0.92 – 1.03	0.398	0.97	0.92 – 1.03	0.317

Note. Associations from separate mixed-effects logistic regression models using predictors for location variance and travel time to predict suicidal event outcomes. Suicidal Events= attempts, psychiatric hospitalizations, and/or emergency department visits for STB concerns. Location variance and travel time (within) indicate person-mean-centered and z-scored variables, such that OR reflected a 1SD increase above one's mean. Location variance and travel time (between) indicate z-scored participant means, such that ORs reflect differences between a participant at the grand mean and a participant 1SD > the grand mean. Time T models indicate concurrent models with geolocation features aggregated the same week as suicidal events, while $Time_{T-1}$ models use geolocation features in the week prior to suicidal events. All models included a covariate for the number of weeks since the baseline visit. *p*-values<.05 are bolded.

© 2025 Auerbach RP et al. *JAMA Network Open*.

eTable 9. Exploration of Associations Between Additional Geolocation Features and Suicidal Ideation

	Time T			Time T			Time T-1			Time T-1		
Predictors	*Odds Ratios*	*95% CI*	*p*	*Odds Ratios*	*95% CI*	*p*	*Odds Ratios*	*95% CI*	*p*	*Odds Ratios*	*95% CI*	*p*
Intercept	---	---	---	---	---	---	---	---	---	---	---	---
Location Variance (Within)	0.90	0.67 – 1.20	0.467				0.95	0.71 – 1.26	0.718			
Location Variance (Between)	0.73	0.39 – 1.36	0.319				0.74	0.40 – 1.35	0.328			
Travel Time (Within)				1.04	0.74 – 1.45	0.827				1.09	0.80 – 1.48	0.604
Travel Time (Between)				0.65	0.31 – 1.35	0.248				0.72	0.34 – 1.51	0.383
Weeks Since Baseline	0.96	0.92 – 1.00	**0.031**	0.96	0.93 – 1.00	0.060	0.97	0.93 – 1.01	0.131	0.96	0.93 – 1.00	0.066

Note. Associations from separate mixed-effects logistic regression models using predictors for location variance and travel time to predict suicidal ideation outcomes. Suicidal Ideation: Experience sampling STB prompts wherein suicidal ideation scores were ≥4 or suicide plans were 'yes'. Location variance and travel time (within) indicate person-mean-centered and z-scored variables, such that OR reflected a 1SD increase above one's mean. Location variance and travel time (between) indicate z-scored participant means, such that ORs reflect differences between a participant at the grand mean and a participant 1SD > the grand mean. Time T models indicate concurrent models with geolocation features aggregated the same week as suicidal ideation, while $Time_{T-1}$ models use geolocation features in the week prior to suicidal ideation. All models included a covariate for the number of weeks since the baseline visit. *p*-values<.05 are bolded.

eFigure 1. Distributions of Participant-level Means of GPS Smartphone Metrics

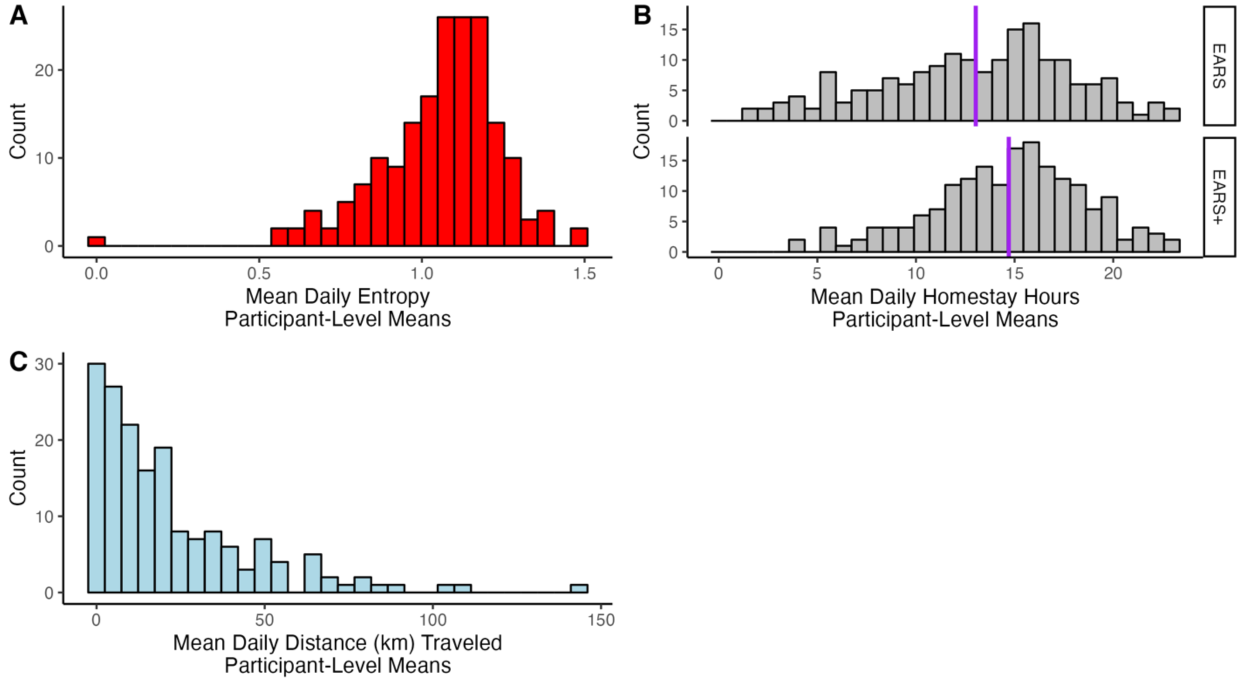

Note. Histograms show participant-level averages of entropy (**A),** homestay (**B)**, and distance traveled **(C)** features derived from smartphone GPS measurements. Features are not standardized or scaled. For **Panel B,** Homestay (EARS)=default EARS home location used for homestay calculation whereas Homestay (EARS+)=multiple home locations included for homestay calculation. Purple lines indicate distribution means.

© 2025 Auerbach RP et al. *JAMA Network Open*.

eFigure 2. Distributions for Weekly Aggregates of Participant-level Means of GPS Smartphone Metrics

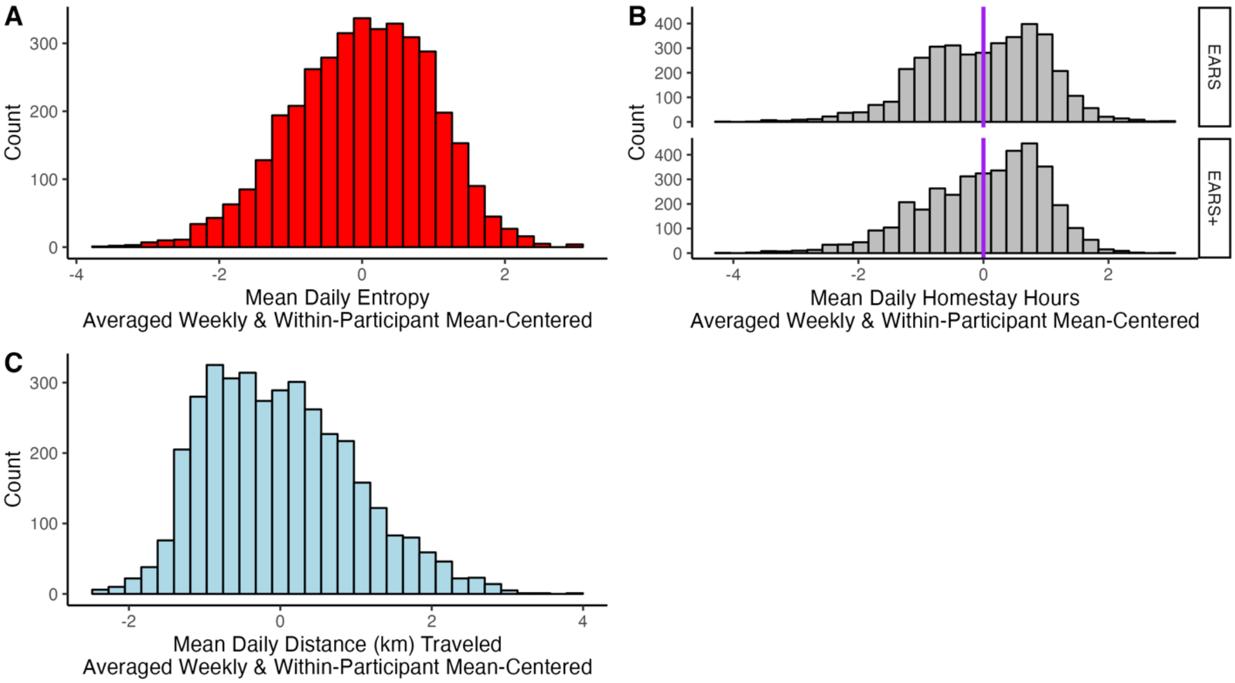

Note. Histograms show weekly aggregates of within-participant mean-centered entropy (**A),** homestay (**B)**, and distance traveled **(C)** features derived from smartphone GPS measurements. Daily geolocation features were first averaged within each week period, then mean-centered for each participant (such that the mean value for each participant is 0), then grand-mean scaled (such that the standard deviation of each distribution is 1). For **Panel B,** Homestay (EARS)=default EARS home location used for homestay calculation whereas Homestay (EARS+)=multiple home locations included for homestay calculation. Purple lines indicate distribution means.

© 2025 Auerbach RP et al. *JAMA Network Open*.

eFigure 3. Missingness of GPS Data and Weekly STB Surveys

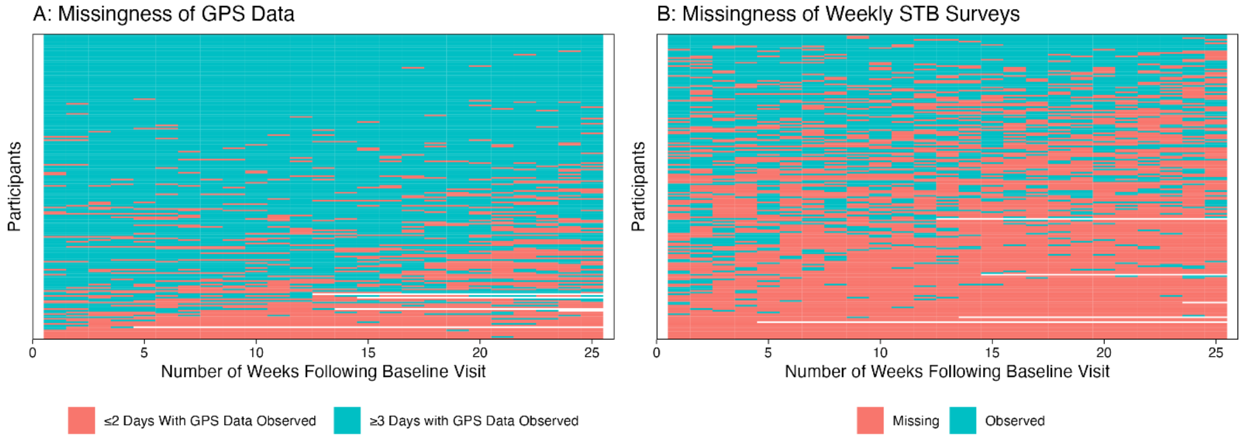

Note. **Panel A:** Missingness of GPS data. For each week following baseline (x axis) for each participant (participants are rows on the y-axis), blue indicates weeks where GPS data was available for 3 or more days and red indicates weeks where GPS data was available for 2 or fewer days (these weeks were excluded from primary analyses). **Panel B:** Missingness of weekly STB surveys conducted through EARS. Blue indicates weeks where a survey response was observed, and red indicates weeks with no response. Weeks missing STB survey data were excluded from primary analyses of suicidal ideation outcomes, but included in primary analyses of suicide event outcomes, as measurement of suicide event outcomes did not depend solely on the weekly STB surveys.

© 2025 Auerbach RP et al. *JAMA Network Open*.

eFigure 4. Timing of Suicidal Events and Suicidal Ideation

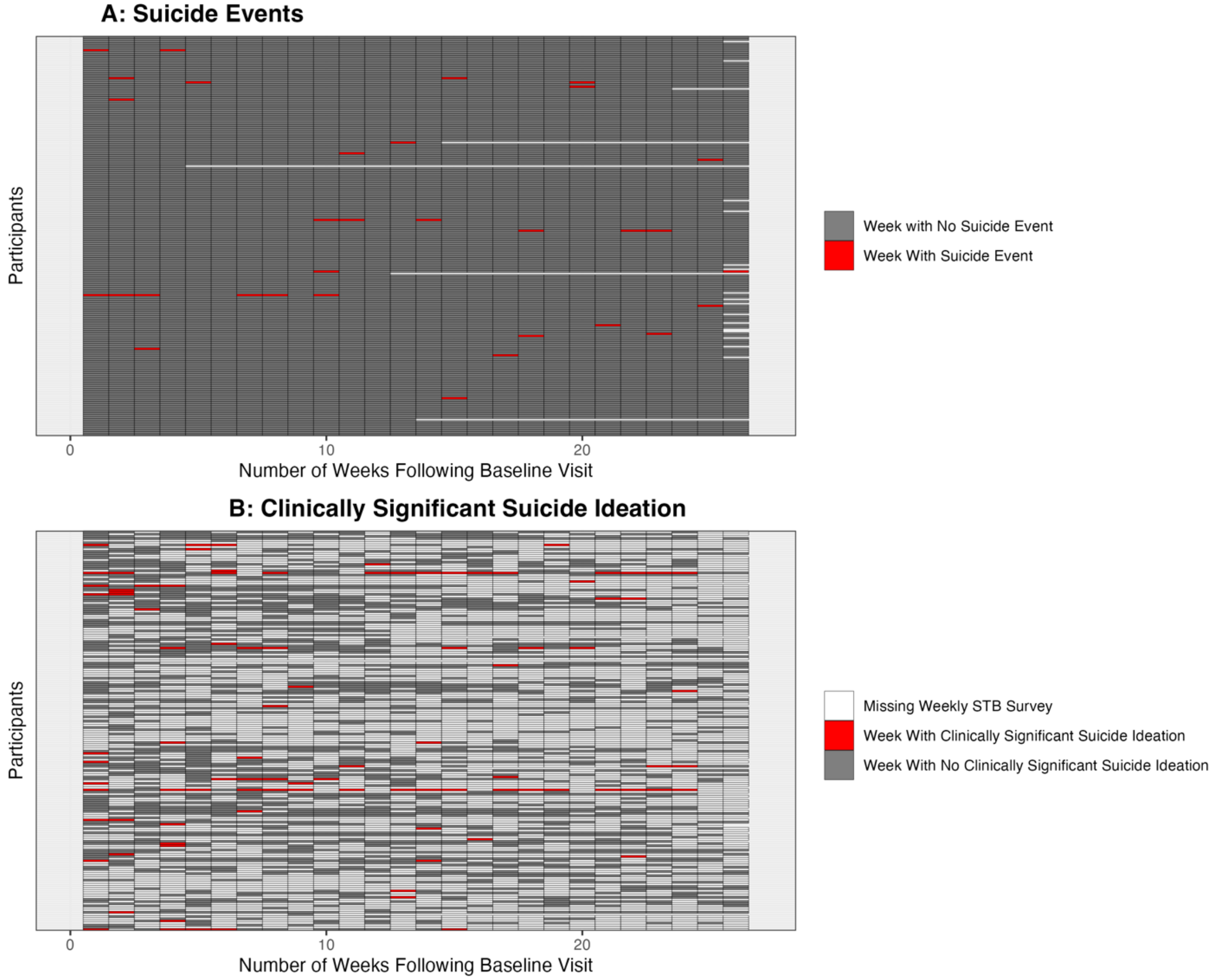

Note. Timing of weeks with suicide events (**Panel A**) and clinically significant suicidal ideation (**Panel B**) are shown, with time following baseline on the x-axis and participants as each row of the y-axis. Red cells indicate suicide events or clinically significant suicidal ideation (EARS survey indicating weekly suicide ideation frequency response ≥4 or "yes" to suicide plans). In **Panel A**, white spaces reflect participants who dropped out before the full 6-month study window (except for the column furthest to the right, which reflects variability in the timing of final follow-up visit; e.g. 26 vs. 27 weeks).

© 2025 Auerbach RP et al. *JAMA Network Open*.

eFigure 5. Within-participant Correlations between Geolocation Measures

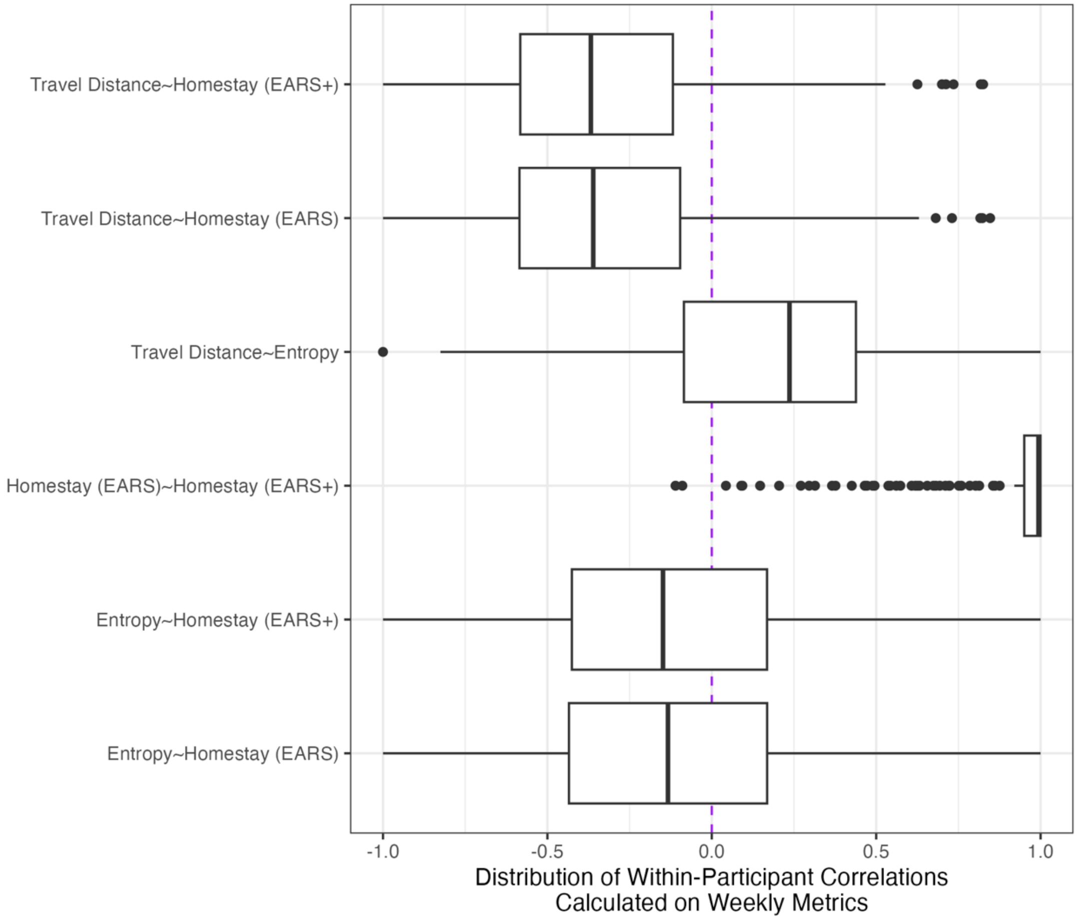

Note. Boxplots show distributions of within-participant correlations between geolocation measures. Correlations are calculated on geolocation metrics averaged within each week as used in primary analyses. Homestay (EARS)=default EARS home location used for homestay calculation whereas Homestay (EARS+)=multiple home locations included for homestay calculation

© 2025 Auerbach RP et al. *JAMA Network Open*.

eFigure 6. Within-person Associations between Homestay Variables and Suicidal Ideation at Different Time Windows

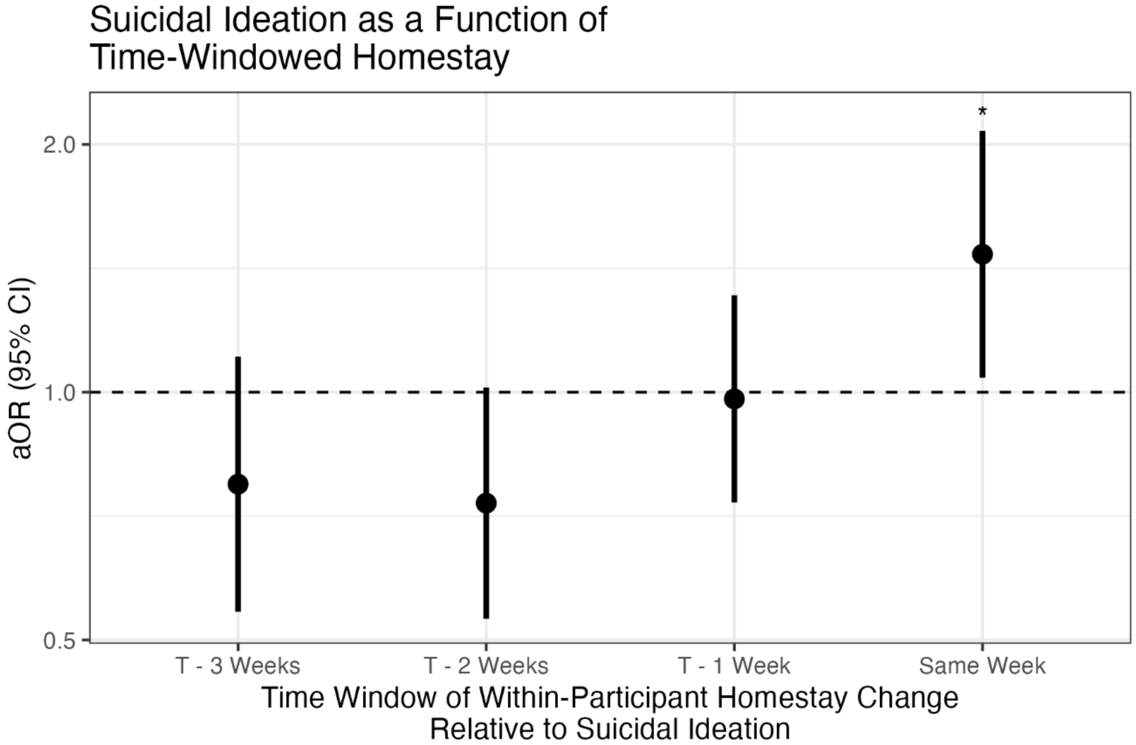

Note. Within-person associations between homestay variables and suicidal ideation at different time windows. Odds ratios (y-axis) and 95% CI for within-person associations between homestay at different time windows and suicidal ideation, ranging from homestay aggregated for week Time_{T-3} to the same week. Significant positive associations between homestay and suicidal ideation were observed for the same week only.

© 2025 Auerbach RP et al. *JAMA Network Open*.

eFigure 7. Within-person and Between-person Associations between Geolocation Features and Suicide Events

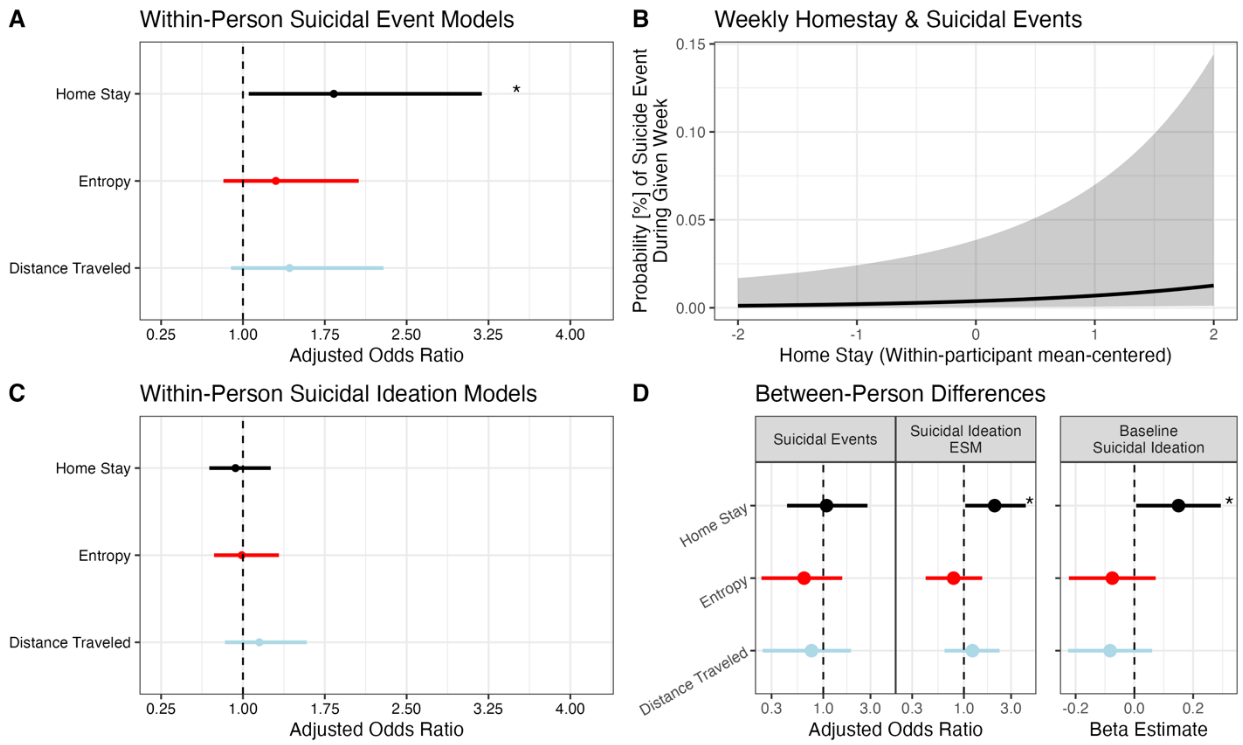

Within-person (**A-C**) and between-person (**D**) associations between geolocation features and suicide events using the EARS+ homestay calculation including multiple possible home locations (parallel to main manuscript **Figure 1)**. **Panel A:** Adjusted odds ratios and 95% CI for within-person associations between geolocation features (black=homestay, red=entropy, blue=distance traveled) and suicide events the following week from multilevel models. All geolocation features were within-participant centered then standardized, such that odds ratios represent changes in odds of suicide events in a given week given a 1SD within-participant increase in the respective geolocation feature. **Panel B:** Estimated probability of suicide events (y-axis) as a function of differences in hours of homestay from a given participant's mean (x-axis). The shaded gray area indicates 95% CI. Note: the y-axis is expressed as a percentage (all predictions indicate <1% likelihood of events). **Panel C:** Adjusted odds ratios and 95% CI for within-person associations between geolocation features and suicidal ideation the next week. **Panel D:** Between-person associations between geolocation features and STB. The left two panes (Suicidal Events, Suicidal Ideation ESM) show between-participants estimates from mixed-effects multilevel regression models predicting same-week outcomes. The right panel (Baseline Suicidal Ideation) shows terms from separate linear regression models between Baseline Suicidal Ideation [SSI] and mean geolocation features. Stars denote associations with $p <$ .05.

© 2025 Auerbach RP et al. *JAMA Network Open*.

eFigure 8. Associations between Geolocation Variables and Suicidal Events at Different Time Windows

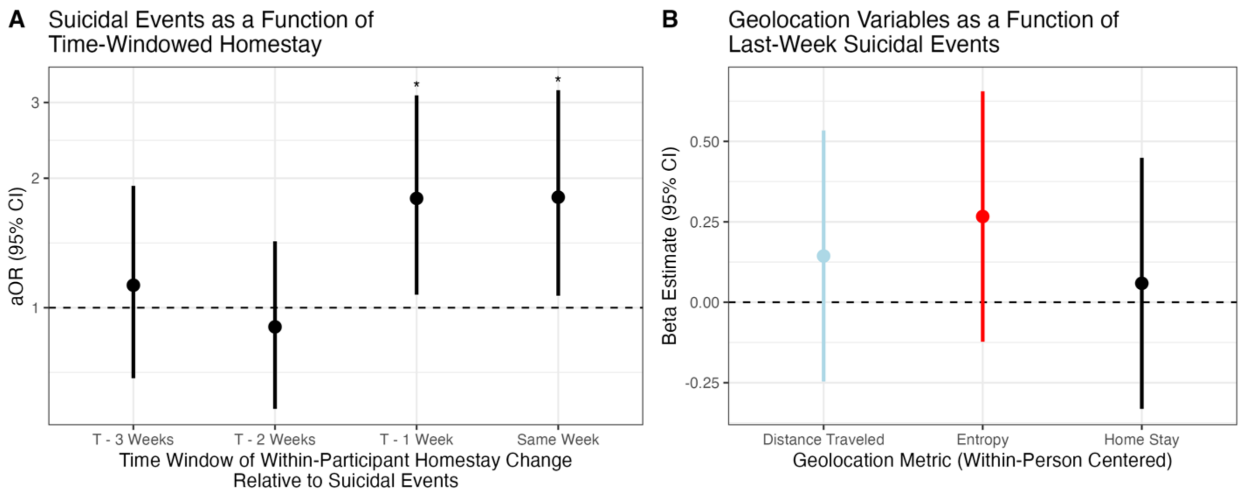

Associations between geolocation variables and suicide events at different time windows using the EARS+ homestay calculation including multiple possible home locations (parallel to main manuscript **Figure 2)**. **Panel A:** Odds ratios (y-axis) and 95% CI for within-person associations between homestay at different time windows and suicide events, ranging from homestay aggregated for week $Time_{T-3}$ to the same week. Significant positive associations between homestay and suicide events were observed for week $Time_{T-1}$ and the same week only. **Panel B:** Beta estimates and 95% for within-person associations between suicide events ($Time_{T-1}$) and geolocation variables the following week ($Time_{T}$). No significant associations between suicide events and geolocation variables the following week were observed. Stars denote associations with $p < .05$.

© 2025 Auerbach RP et al. *JAMA Network Open*.

eFigure 9. Associations between Geolocation Variables and Suicidal Events the Following Week with Sensitivity Checks

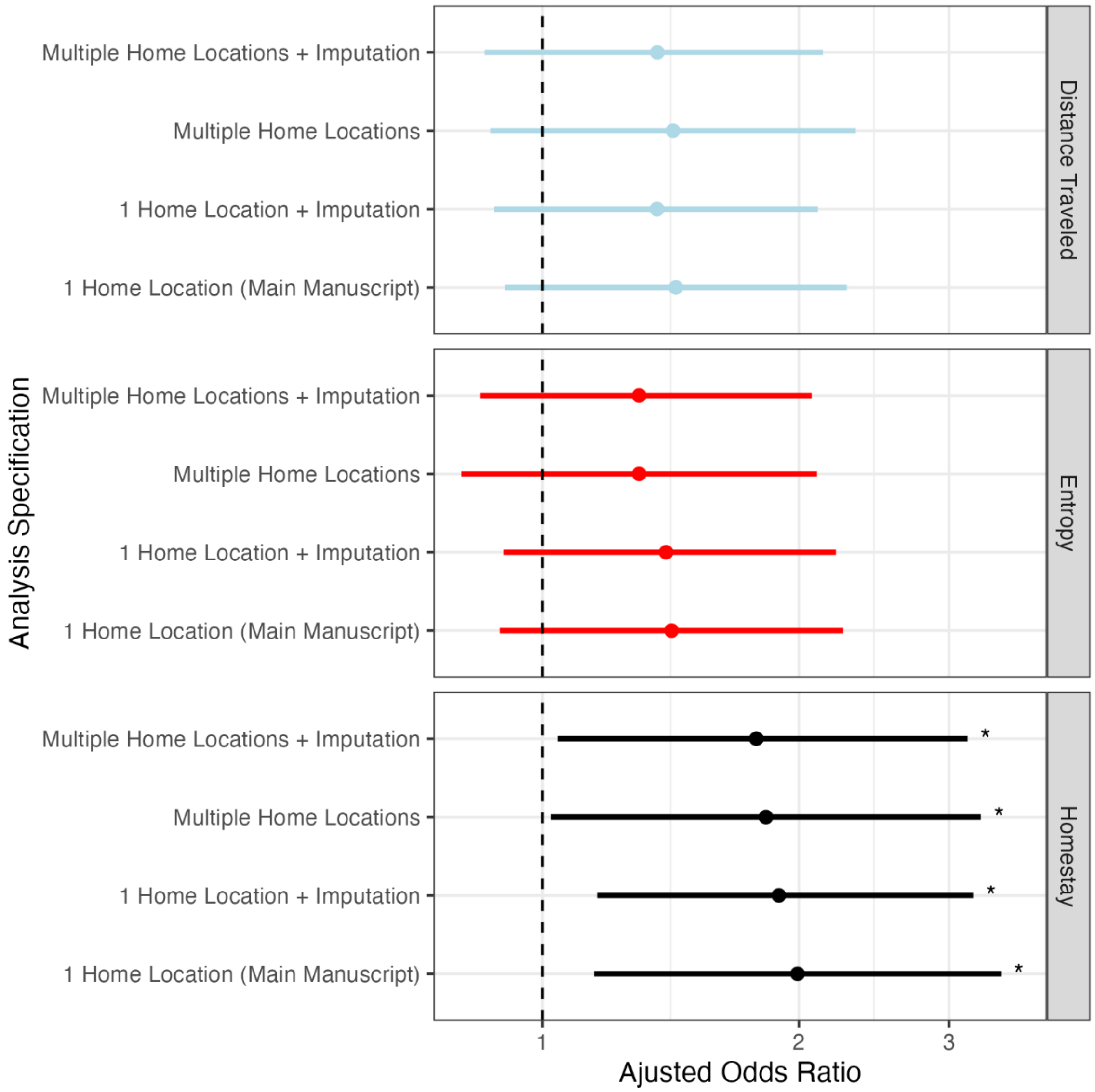

Associations between geolocation variables and suicide events the following week under sensitivity checks a & b for home locations and imputation strategy. The model reported in the main manuscript is also visualized for comparison. Adjusted odds ratios (x-axis) and 95% CI for within person associations between geolocation features (top panel=distance traveled, middle=entropy, bottom=homestay) aggregated at $Time_{T-1}$ and suicide events are shown. Y-axis labels describe sensitivity check specifications. Multiple Home Locations=use of the EARS+ homestay calculation including multiple possible home locations, 1 Home Location=use of the default EARS homestay calculation using just one home location, Imputation=model was fit using joint analysis and imputation, Main Manuscript indicates the model reported in the main manuscript. Stars denote associations with $p < .05$.

© 2025 Auerbach RP et al. *JAMA Network Open*.

eFigure 10. Within-person and Between-person Associations between Geolocation Features and Suicidal Events Using the Recoded Timing

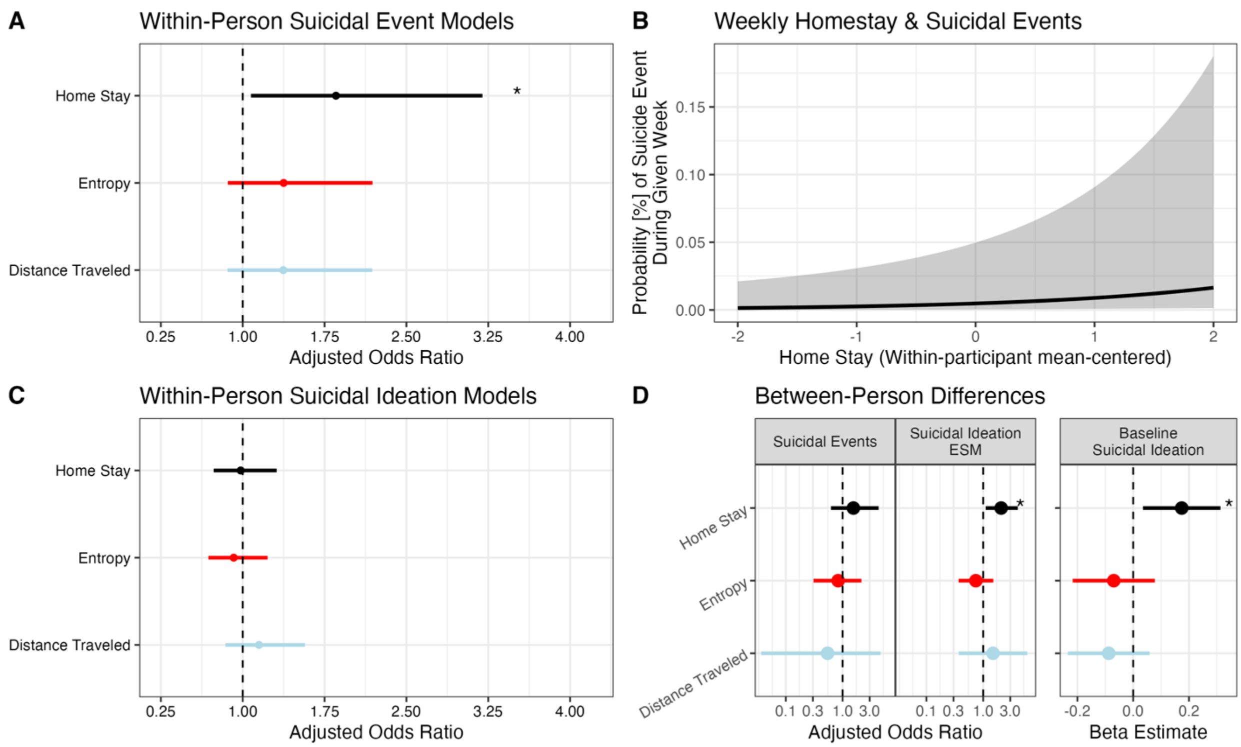

Within-person (**A-C**) and between-person (**D**) associations between geolocation features and suicide events using the recoded suicidal event timing described in sensitivity check e. (parallel to main manuscript **Figure 1**). **Panel A:** Adjusted odds ratios and 95% CI for within-person associations between geolocation features (black=homestay, red=entropy, blue=distance traveled) and suicide events the following week from multilevel models. All geolocation features were within-participant centered then standardized, such that odds ratios represent changes in odds of suicide events in a given week given a 1SD within-participant increase in the respective geolocation feature. **Panel B:** Estimated probability of suicide events (y-axis) as a function of differences in hours of homestay from a given participant's mean (x-axis). The shaded gray area indicates 95% CI. Note: the y-axis is expressed as a percentage (all predictions indicate <1% likelihood of events). **Panel C:** Adjusted odds ratios and 95% CI for within-person associations between geolocation features and suicidal ideation the next week. **Panel D:** Between-person associations between geolocation features and STB. The left two panes (Suicidal Events, Suicidal Ideation ESM) show between-participants estimates from mixed-effects multilevel regression models predicting same-week outcomes. The right panel (Baseline Suicidal Ideation) shows terms from separate linear regression models between Baseline Suicidal Ideation [SSI] and mean geolocation features. Stars denote associations with $p < .05$.

© 2025 Auerbach RP et al. *JAMA Network Open*.

eFigure 11. Associations between Geolocation Variables and Suicidal Events at Different Time Windows Using the Recoded Suicidal Event Timing Described in Sensitivity Check e

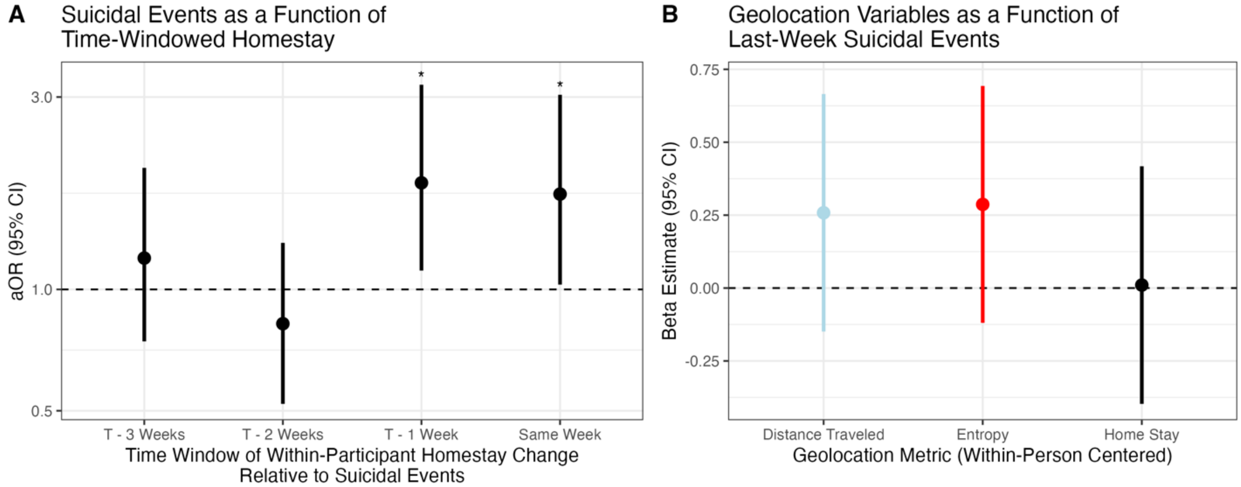

Associations between geolocation variables and suicide events at different time windows using the recoded suicidal event timing described in sensitivity check e. (parallel to main manuscript **Figure 2)**. **Panel A:** Odds ratios (y-axis) and 95% CI for within-person associations between homestay at different time windows and suicide events, ranging from homestay aggregated for week $Time_{T-3}$ to the same week. Significant positive associations between homestay and suicide events were observed for week $Time_{T-1}$ and the same week only. **Panel B:** Beta estimates and 95% for within-person associations between suicide events ($Time_{T-1}$) and geolocation variables the following week ($Time_{T}$). No significant associations between suicide events and geolocation variables the following week were observed. Stars denote associations with p < .05.

© 2025 Auerbach RP et al. *JAMA Network Open*.

eFigure 12. Multiverse Sensitivity Checks for Within-person Associations between Homestay and Next-week Suicidal Events

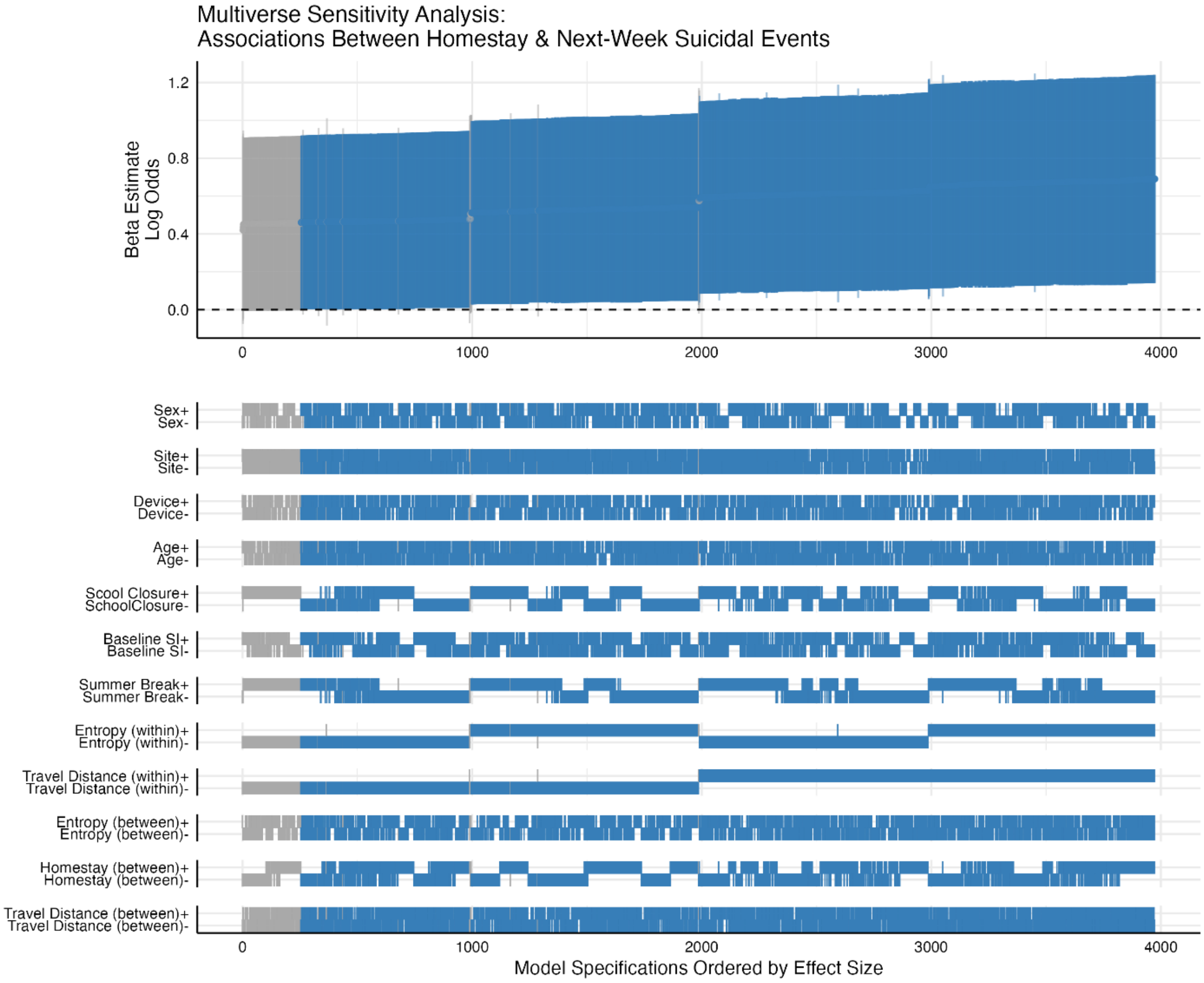

Multiverse sensitivity checks for within-person associations between homestay and next-week suicidal events under different covariate scenarios. **Top Panel:** points and error bars show beta estimates (in log odds) and 95% CI from individual mixed-effects logistic regression models estimating the within-person association between homestay and suicidal events. Positive values indicate a positive association. Models are ordered by the magnitude of beta estimates. Effects are regarded as significant when 95% CI do not overlap 0 (dotted line). **Bottom Panel:** Model specification information corresponding to each estimate in the top panel, where each column is one model. Variables on the y-axis represent covariates, with the + symbol indicating a covariate was included, and the – symbol indicating it was not included in a given model. Across both panels color indicates sign and significance (blue = positive significant, red = negative significant, gray = not significant).

© 2025 Auerbach RP et al. *JAMA Network Open*.

eFigure 13. Multiverse Sensitivity Checks for Within-person Associations between Entropy and Next-week Suicidal Events under Different Covariate Scenarios

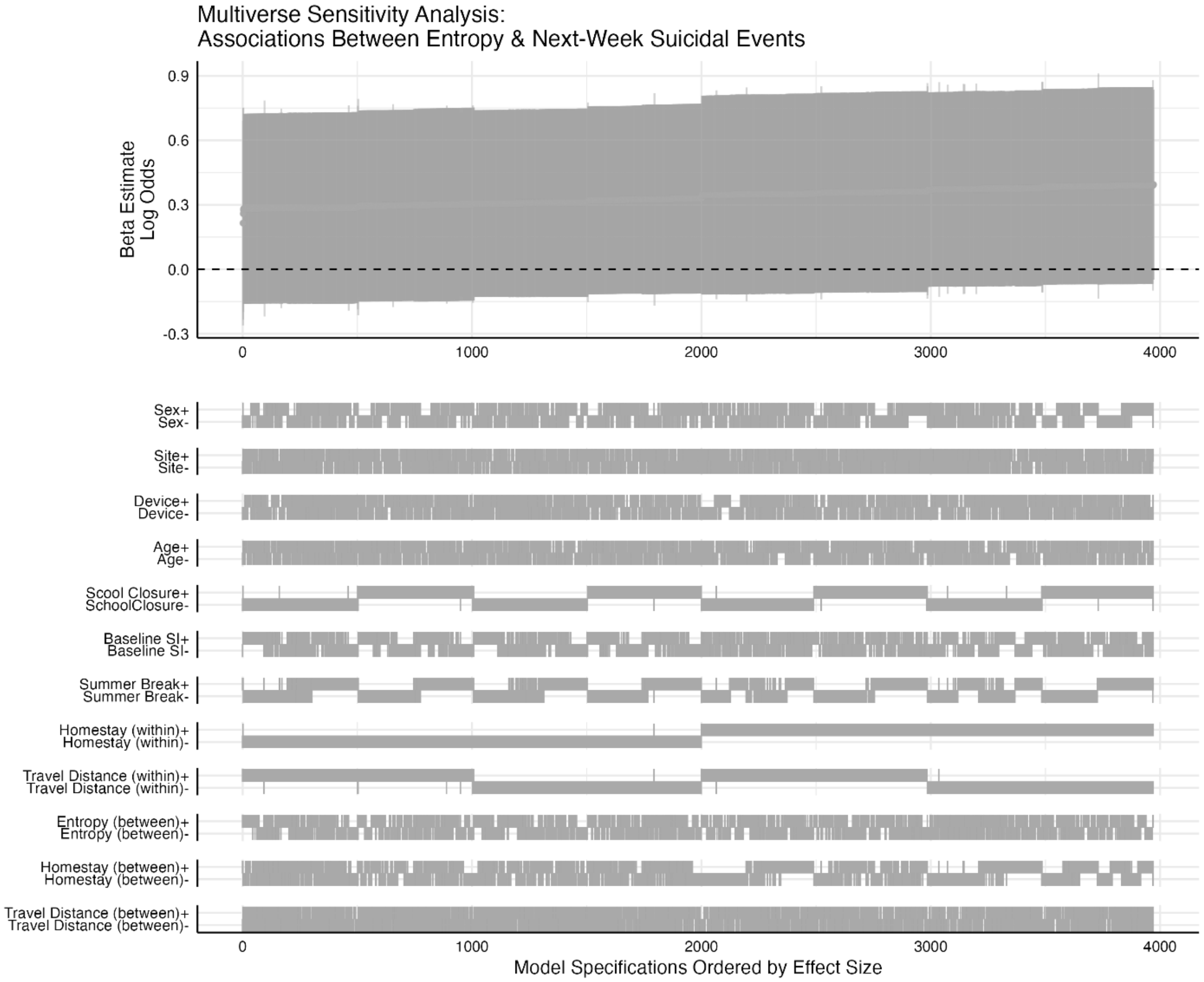

Multiverse sensitivity checks for within-person associations between entropy and next-week suicidal events under different covariate scenarios. **Top Panel:** points and error bars show beta estimates (in log odds) and 95% CI from individual mixed-effects logistic regression models estimating the within-person association between entropy and suicidal events. Positive values indicate a positive association. Models are ordered by the magnitude of beta estimates. Effects are regarded as significant when 95% CI do not overlap 0 (dotted line). **Bottom Panel:** Model specification information corresponding to each estimate in the top panel, where each column is one model. Variables on the y-axis represent covariates, with the + symbol indicating a covariate was included, and the – symbol indicating it was not included in a given model. Across both panels color indicates sign and significance (blue = positive significant, red = negative significant, gray = not significant).

© 2025 Auerbach RP et al. *JAMA Network Open*.

eFigure 14. Multiverse Sensitivity Checks for Within-person Associations between Travel Distance and Next-week Suicidal Events under Different Covariate Scenarios

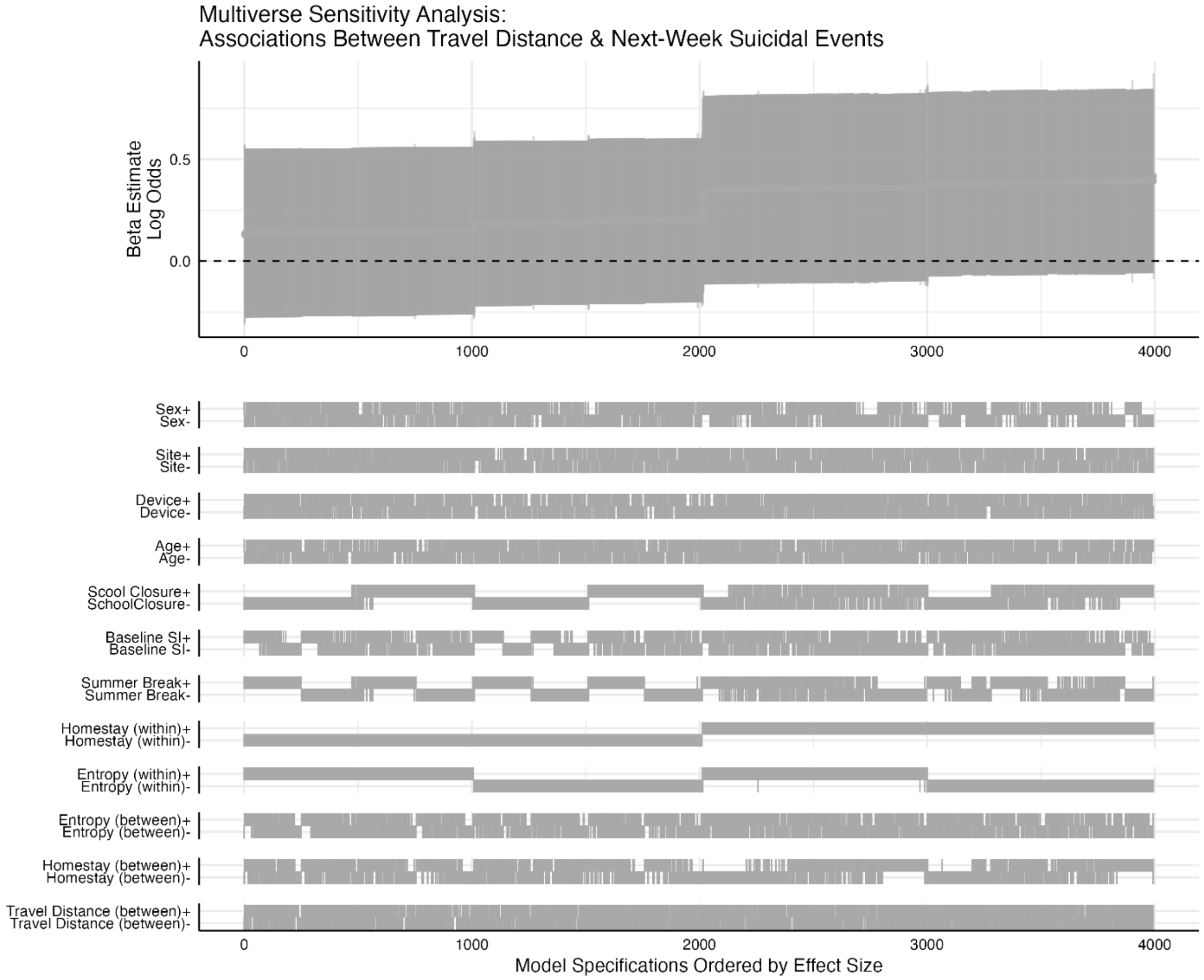

Multiverse sensitivity checks for within-person associations between travel distance and next-week suicidal events under different covariate scenarios. **Top Panel:** points and error bars show beta estimates (in log odds) and 95% CI from individual mixed-effects logistic regression models estimating the within-person association between travel distance and suicidal events. Positive values indicate a positive association. Models are ordered by the magnitude of beta estimates. Effects are regarded as significant when 95% CI do not overlap 0 (dotted line). **Bottom Panel:** Model specification information corresponding to each estimate in the top panel, where each column is one model. Variables on the y-axis represent covariates, with the + symbol indicating a covariate was included, and the – symbol indicating it was not included in a given model. Across both panels color indicates sign and significance (blue = positive significant, red = negative significant, gray = not significant).

eFigure 15. Multiverse Sensitivity Checks for Within-person Associations between Homestay and Next-week Suicidal Ideation under Different Covariate Scenarios

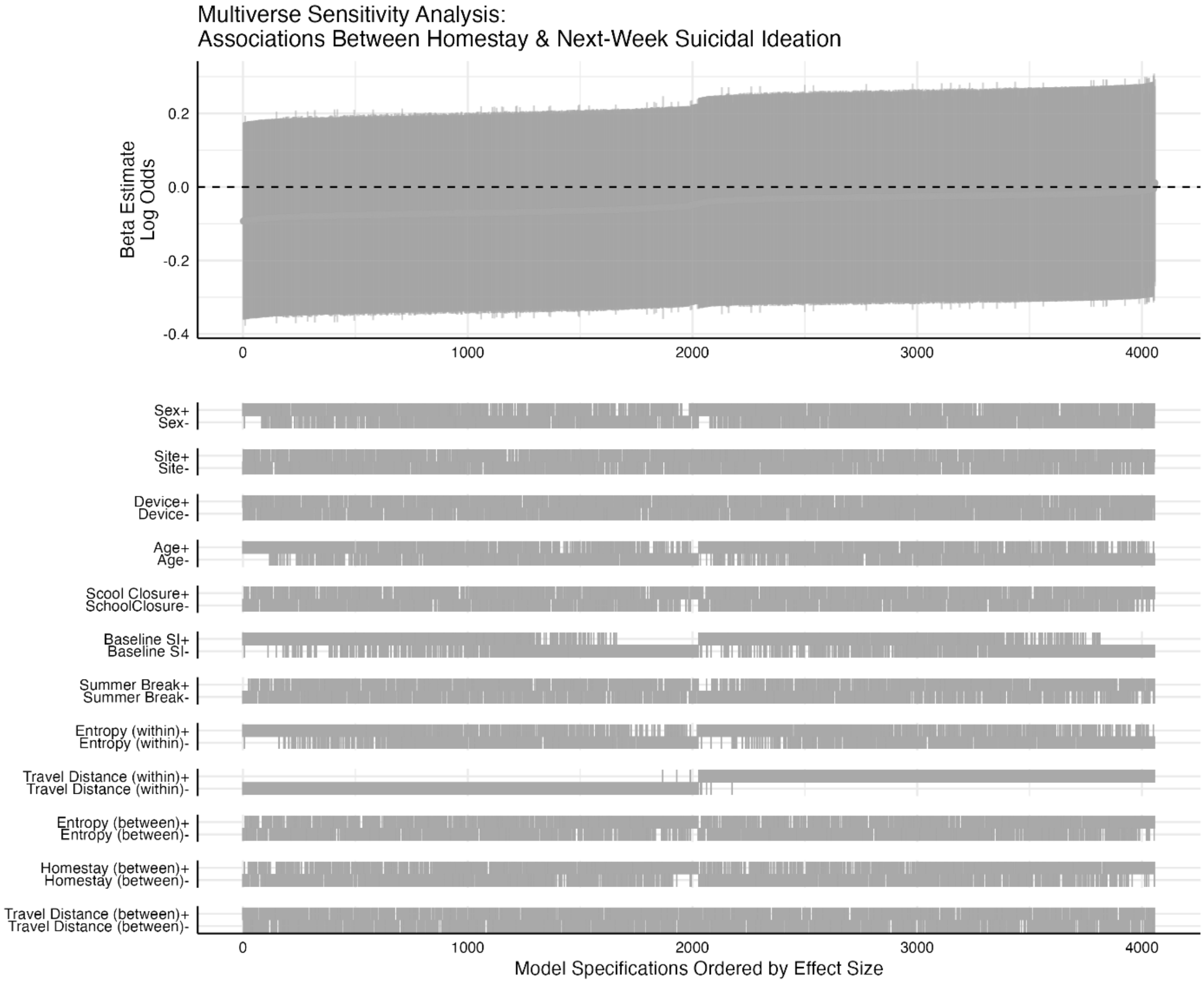

Multiverse sensitivity checks for within-person associations between homestay and next-week suicidal ideation under different covariate scenarios. **Top Panel:** points and error bars show beta estimates (in log odds) and 95% CI from individual mixed-effects logistic regression models estimating the within-person association between homestay and suicidal ideation. Positive values indicate a positive association. Models are ordered by the magnitude of beta estimates. Effects are regarded as significant when 95% CI do not overlap 0 (dotted line). **Bottom Panel:** Model specification information corresponding to each estimate in the top panel, where each column is one model. Variables on the y-axis represent covariates, with the + symbol indicating a covariate was included, and the – symbol indicating it was not included in a given model. Across both panels color indicates sign and significance (blue = positive significant, red = negative significant, gray = not significant).

© 2025 Auerbach RP et al. *JAMA Network Open*.

eFigure 16. Multiverse Sensitivity Checks for Within-person Associations between Entropy and Next-week Suicidal Ideation under Different Covariate Scenarios

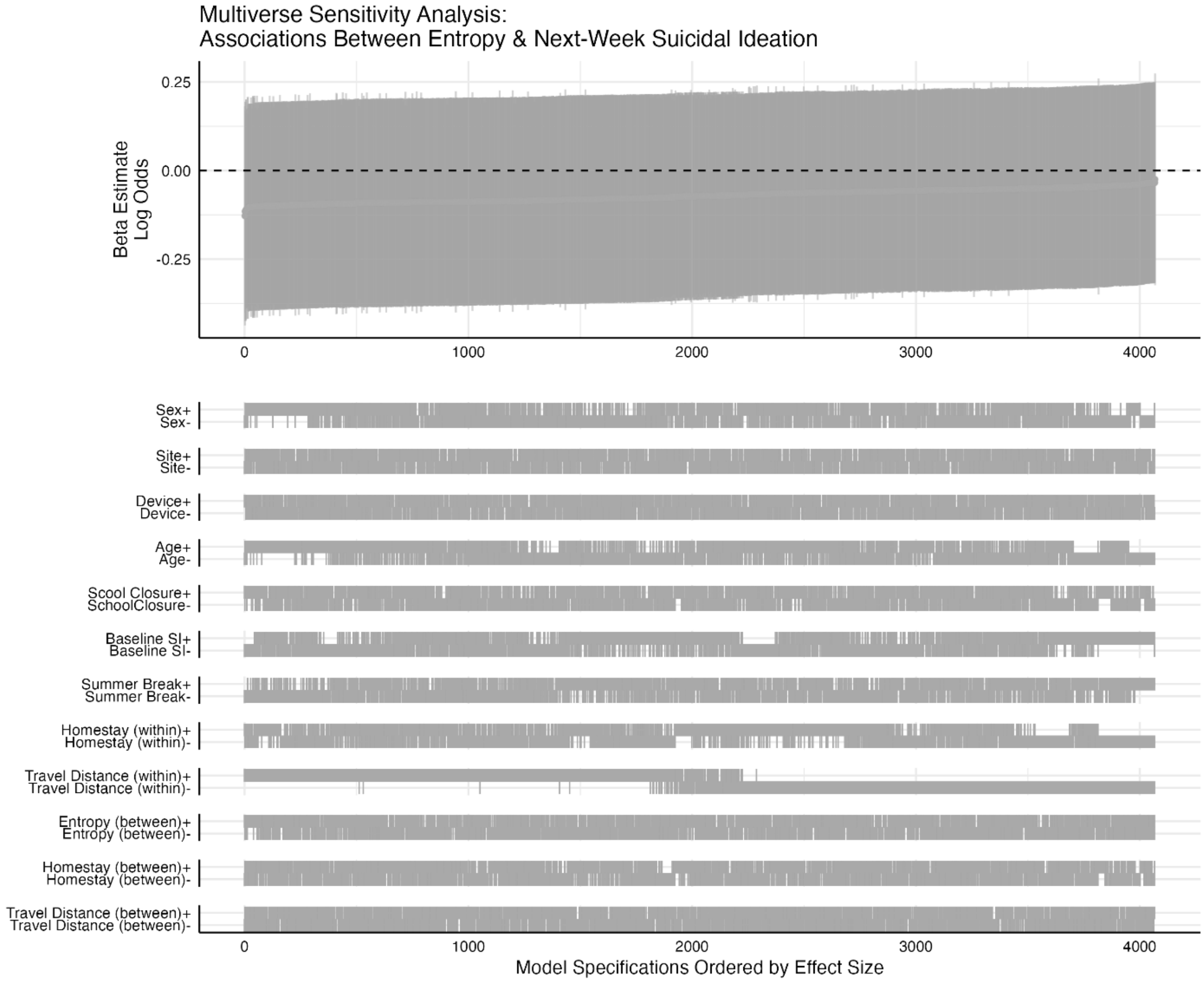

Multiverse sensitivity checks for within-person associations between entropy and next-week suicidal ideation under different covariate scenarios. **Top Panel:** points and error bars show beta estimates (in log odds) and 95% CI from individual mixed-effects logistic regression models estimating the within-person association between entropy and suicidal ideation. Positive values indicate a positive association. Models are ordered by the magnitude of beta estimates. Effects are regarded as significant when 95% CI do not overlap 0 (dotted line). **Bottom Panel:** Model specification information corresponding to each estimate in the top panel, where each column is one model. Variables on the y-axis represent covariates, with the + symbol indicating a covariate was included, and the – symbol indicating it was not included in a given model. Across both panels color indicates sign and significance (blue = positive significant, red = negative significant, gray = not significant).

© 2025 Auerbach RP et al. *JAMA Network Open*.

eFigure 17. Multiverse Sensitivity Checks for Within-person Associations between Travel Distance and Next-week Suicidal Ideation under Different Covariate Scenarios

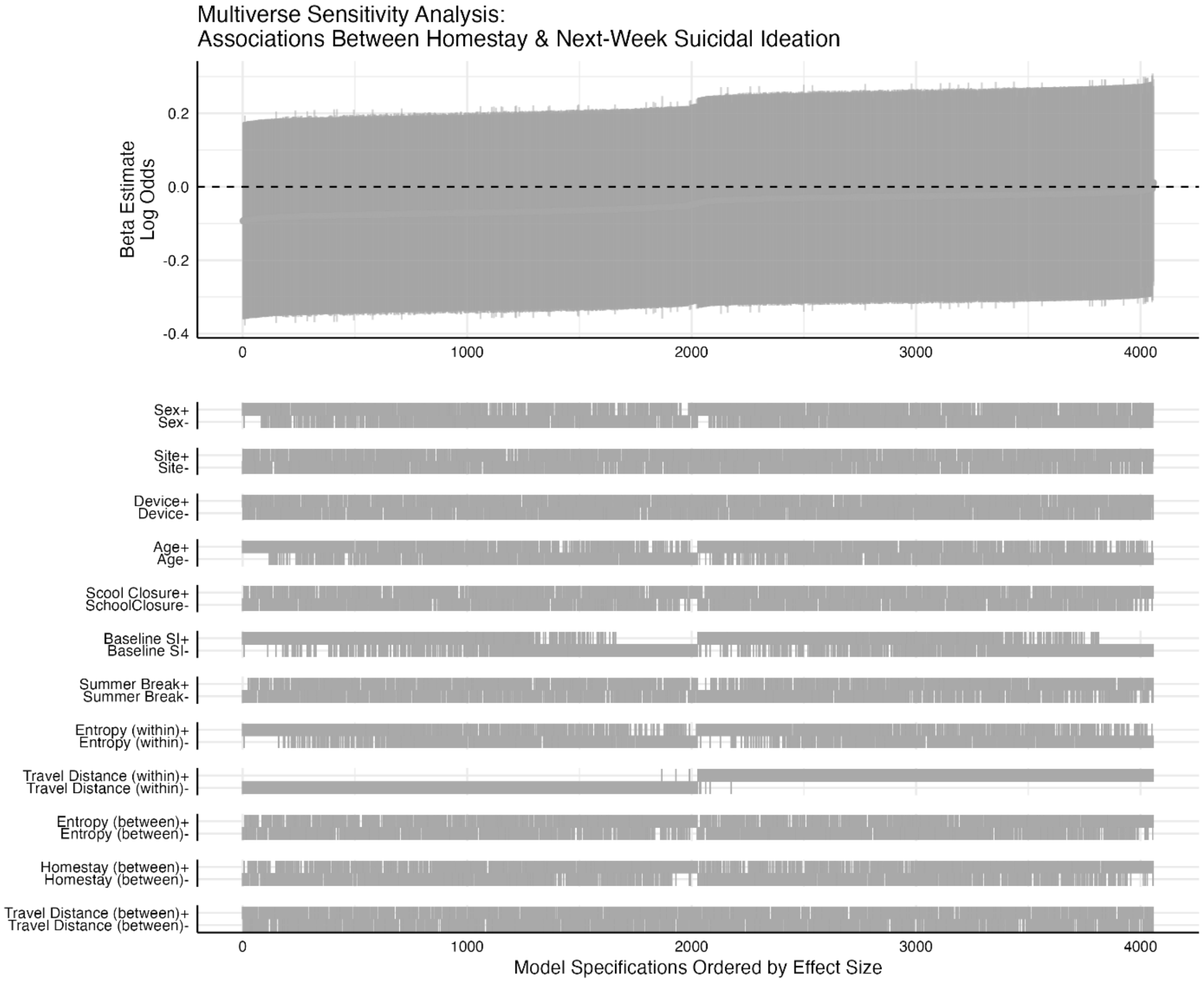

Multiverse sensitivity checks for within-person associations between travel distance and next-week suicidal ideation under different covariate scenarios. **Top Panel:** points and error bars show beta estimates (in log odds) and 95% CI from individual mixed-effects logistic regression models estimating the within-person association between travel distance and suicidal ideation. Positive values indicate a positive association. Models are ordered by the magnitude of beta estimates. Effects are regarded as significant when 95% CI do not overlap 0 (dotted line). **Bottom Panel:** Model specification information corresponding to each estimate in the top panel, where each column is one model. Variables on the y-axis represent covariates, with the + symbol indicating a covariate was included, and the – symbol indicating it was not included in a given model. Across both panels color indicates sign and significance (blue = positive significant, red = negative significant, gray = not significant).

© 2025 Auerbach RP et al. *JAMA Network Open*.

eFigure 18. Evaluation of the Accuracy of Predictive Models of Suicidal Ideation and Events

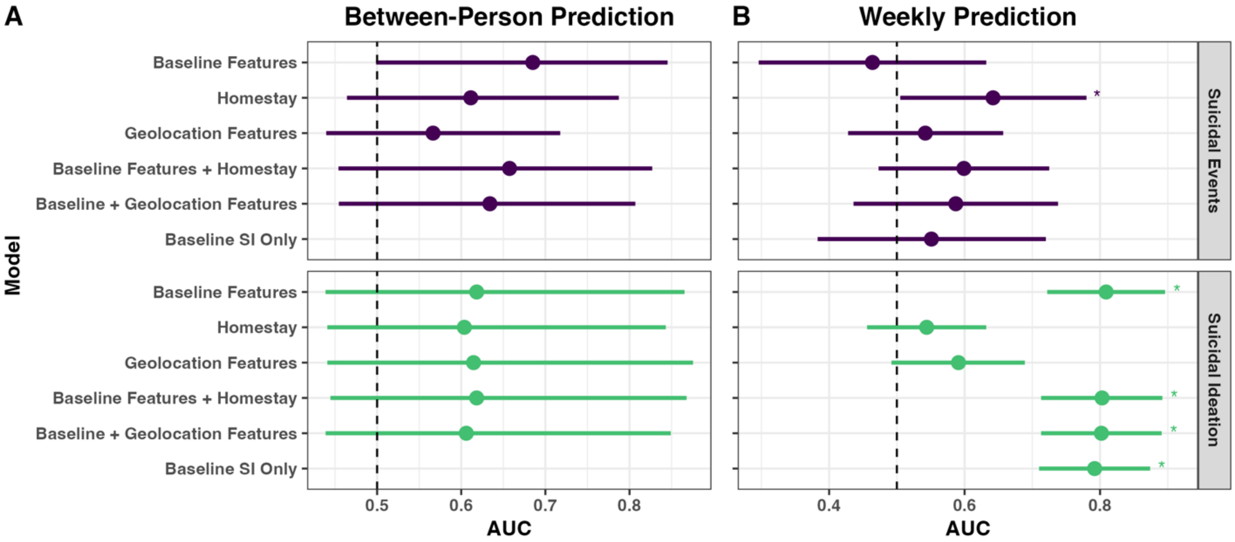

Note. Evaluation of the accuracy of predictive models of suicidal ideation (top) and events (bottom), with an additional model including only Baseline suicidal ideation (Baseline SI Only). **Panel A:** Between-person prediction using of which participants will experience suicidal ideation (top) or events (bottom) during the last 5 months of the study window. X-axis shows AUC scores and 95% CI within the held-out 30% of participants for logistic regression models using geolocation features (homestay, entropy, distance traveled) aggregated over the first month of the study window, only homestay, baseline features (baseline suicidal ideation, sex, site, age, device type), only baseline suicidal ideation, and the combination of geolocation and baseline features. **Panel B:** Within-participant prediction using leave-future-out validation of which weeks participants will experience suicidal ideation (top) or events (bottom). X-axis shows AUC scores and 95% CI within the held-out final half of the study (3-months) for each participant for models paralleling those in Panel A, but with time-varying within-participant weekly geolocation features. Stars denote above-chance performance (lower bound of 95% CI >= 0.5). As an external dataset was not available for validation analyses, model predictive performance metrics based on internal validation may be inflated.

eReferences

1. Sheehan DV. *Mini International Neuropsychiatric Interview for Children and Adolescents English version 7.0.2*. Medical Outcome Systems; 2016.
2. Nock MK, Holmberg EB, Photos VI, Michel BD. Self-Injurious Thoughts and Behaviors Interview: development, reliability, and validity in an adolescent sample. 2007;
3. Posner K, Brown GK, Stanley B, et al. The Columbia–Suicide Severity Rating Scale: initial validity and internal consistency findings from three multisite studies with adolescents and adults. *American journal of psychiatry*. 2011;168(12):1266-1277.
4. Erler NS, Rizopoulos D, Lesaffre EMEH. JointAI: Joint Analysis and Imputation of Incomplete Data in R. *Journal of Statistical Software*. 2021;100(20):1-56. doi:10.18637/jss.v100.i20
5. Steegen S, Tuerlinckx F, Gelman A, Vanpaemel W. Increasing Transparency Through a Multiverse Analysis. *Perspect Psychol Sci*. 2016;11(5):702-712. doi:10.1177/1745691616658637
6. Bürkner PC. brms: Bayesian Regression Models using “Stan.” Published online August 29, 2019. https://CRAN.R-project.org/package=brms

© 2025 Auerbach RP et al. *JAMA Network Open*.
