# Supplementary material for: Using Smartphone GPS Data to Detect the Risk of Adolescent Suicidal Thoughts and Behaviors
Source: JAMA Netw Open. 2025 Jan 27;8(1):e2456429. doi: 10.1001/jamanetworkopen.2024.56429 (PMC11773992; doi:10.1001/jamanetworkopen.2024.56429)
Supplement: Supplement 2. — Data Sharing Statement [file jamanetwopen-e2456429-s002.pdf]

Data Sharing Statement

Auerbach. Using Smartphone GPS Data to Improve the Prediction of Adolescent Suicidal Thoughts and Behaviors. *JAMA Netw Open*. Published January 27, 2025. doi:10.1001/jamanetworkopen.2024.56429

Data
Data available: Yes
Data types: Deidentified participant data, Data dictionary
How to access data: All data is available through the National Data Archive
When available: beginning date: 01-15-2024

Supporting Documents
Document types: None

Additional Information
Who can access the data: All data and the data dictionary are available through the NDA per NIMH guidelines.
Types of analyses: All data and the data dictionary are available through the NDA per NIMH guidelines.
Mechanisms of data availability: All data and the data dictionary are available through the NDA per NIMH guidelines.
